# Identification of distinct clinical phenotypes of acute respiratory distress syndrome with differential responses to treatment

**DOI:** 10.1186/s13054-021-03734-y

**Published:** 2021-08-30

**Authors:** Xiaowei Liu, Yusheng Jiang, Xiaonan Jia, Xiaohui Ma, Ci Han, Nana Guo, Yahui Peng, Haitao Liu, Yingnan Ju, Xiangfeng Luo, Xueting Li, Yue Bu, Jin Zhang, Yansong Liu, Yan Gao, Mingyan Zhao, Hongliang Wang, Ligang Luo, Kaijiang Yu, Changsong Wang

**Affiliations:** 1 Department of Critical Care Medicine, First Affiliated Hospital of Harbin Medical University, 23 Postal Street, Nangang District, Harbin, 150001 Heilongjiang China; 2 LinkDoc AI Lab, LinkDoc Technology, Floor 11, Sinosteel Plaza, 8 Haidian Street, Haidian District, Beijing, 100080 China; 3 Department of Critical Care Medicine, Harbin Medical University Cancer Hospital, 150 Haping Road, Nangang District, Harbin, 150081 Heilongjiang China; 4 Department of Critical Care Medicine, Forth Affiliated Hospital of Harbin Medical University, 37 Yiyuan Street, Nangang District, Harbin, 150001 Heilongjiang China; 5 Department of Critical Care Medicine, Second Affiliated Hospital of Harbin Medical University, 246 Xuefu Road, Nangang District, Harbin, 150001 Heilongjiang China

**Keywords:** Acute respiratory distress syndrome, Phenotype, Cluster analysis, Clinical characteristics and outcomes, Treatment strategy

## Abstract

**Background:**

Acute respiratory distress syndrome (ARDS) is a heterogeneous syndrome, and the identification of homogeneous subgroups and phenotypes is the first step toward precision critical care. We aimed to explore whether ARDS phenotypes can be identified using clinical data, are reproducible and are associated with clinical outcomes and treatment response.

**Methods:**

This study is based on a retrospective analysis of data from the telehealth intensive care unit (eICU) collaborative research database and three ARDS randomized controlled trials (RCTs) (ALVEOLI, FACTT and SAILS trials). We derived phenotypes in the eICU by cluster analysis based on clinical data and compared the clinical characteristics and outcomes of each phenotype. The reproducibility of the derived phenotypes was tested using the data from three RCTs, and treatment effects were evaluated.

**Results:**

Three clinical phenotypes were identified in the training cohort of 3875 ARDS patients. Of the three phenotypes identified, phenotype I (*n* = 1565; 40%) was associated with fewer laboratory abnormalities, less organ dysfunction and the lowest in-hospital mortality rate (8%). Phenotype II (*n* = 1232; 32%) was correlated with more inflammation and shock and had a higher mortality rate (18%). Phenotype III (*n* = 1078; 28%) was strongly correlated with renal dysfunction and acidosis and had the highest mortality rate (22%). These results were validated using the data from the validation cohort (*n* = 3670) and three RCTs (*n* = 2289) and had reproducibility. Patients with these ARDS phenotypes had different treatment responses to randomized interventions. Specifically, in the ALVEOLI cohort, the effects of ventilation strategy (high PEEP vs low PEEP) on ventilator-free days differed by phenotype (*p* = 0.001); in the FACTT cohort, there was a significant interaction between phenotype and fluid-management strategy for 60-day mortality (*p* = 0.01). The fluid-conservative strategy was associated with improved mortality in phenotype II but had the opposite effect in phenotype III.

**Conclusion:**

Three clinical phenotypes of ARDS were identified and had different clinical characteristics and outcomes. The analysis shows evidence of a phenotype-specific treatment benefit in the ALVEOLI and FACTT trials. These findings may improve the identification of distinct subsets of ARDS patients for exploration in future RCTs.

**Supplementary Information:**

The online version contains supplementary material available at 10.1186/s13054-021-03734-y.

## Introduction

According to the Berlin definition, acute respiratory distress syndrome (ARDS) is a clinical syndrome defined by acute-onset hypoxemia (partial pressure of arterial oxygen [PaO2] to fraction of inspired oxygen [FiO2] ratio < 300) and bilateral pulmonary opacities not fully explained by cardiac failure or volume overload [1]. A wide variety of etiologies and pathologies are included in this definition, leading to complex biological and clinical heterogeneity [2]. This heterogeneity is recognized as the main contributor to negative treatment outcomes for ARDS in numerous randomized controlled trials (RCTs) with regard to pharmacological interventions [3, 4]. In recent decades, positive results of trials of several supportive care interventions, including lung protective mechanical ventilation [5], neuromuscular blockade [6] and prone positioning [7], have led to improvements in ARDS survival. However, ARDS is still a major complication in critically ill patients and has high morbidity and mortality rates [8–10]. Consequently, it is important to identify distinct subgroups of ARDS patients and increase the efficacy of interventions with targeted subgroups.

Our understanding of the heterogeneity of critical illness syndromes has improved with the use of mathematical and statistical methods, such as cluster analysis and latent class analysis (LCA) [11]. Seymour and colleagues identified four clinical phenotypes of sepsis by cluster analysis, and they were correlated with host-response patterns and clinical outcomes [12]. Similarly, two ARDS subphenotypes (hyperinflammatory and hypoinflammatory) have been identified from analyses of four cohorts of patients derived from the National Heart, Lung, and Blood Institute (NHLBI) ARDS Network RCT [13–15]. They had different mortality rates and different treatment responses to randomized interventions. However, all these ARDS studies used plasma biomarkers as class-defining variables, such as sTNFR-1 and interleukins (ILs), which are not routinely available and cannot be quantified rapidly at the bedside. Thus, the clinical applicability of this classification system may be limited.

In this study, we hypothesized that more phenotypes could be identified by cluster analysis based on routine clinical data in a large ARDS target population and that these phenotypes were associated with clinical outcomes. In addition, the derived phenotypes were assessed in other ARDS validation cohorts. Furthermore, we hypothesized that these ARDS phenotypes were associated with different treatment responses to randomized interventions.

## Methods

### Study design

Our study included one large database and three RCTs (Additional file 1: Figure S1). First, we derived clinical phenotypes by cluster analysis in the derivation cohort. A clustering model was trained in this step. Second, we cross-validated the clustering analysis results in the validation cohort and tested their stability. Third, we assessed the reproducibility of the derived ARDS phenotypes using the data from three RCTs. Fourth, we compared the treatment effects between phenotypes using the data from three RCTs. Finally, the derived clinical phenotypes were compared with traditional patient risk stratification metrics, such as the Berlin classification of ARDS and Acute Physiology and Chronic Health Evaluation (APACHE) score.

### Population

The analysis is based on the telehealth intensive care unit (eICU) collaborative research database, a multicenter ICU database with over 200,000 electronic medical records from 335 units at 208 hospitals across the USA [16]. We used the International Classification of Diseases, Ninth Revision, Clinical Modification (ICD-9-CM) codes and APACHE Admission Diagnosis entry to identify patients who met the ARDS diagnostic criteria. Patients were excluded based on a set of exclusion criteria, for example, expiration of patients within 24 h, disqualifying the P/F ratio, or missing over half of the clinical variables (SMethods and Additional file 1: Figure S2). Patients discharged from the hospital in 2014 were enrolled in the training cohort, and those discharged in 2015 were enrolled in the validation cohort.

All three RCTs were multicenter studies from the ARDS Network, funded by the NHLBI, and included the ALVEOLI, FACTT and SAILS trials [17–19]. The ALVEOLI trial was a multicenter randomized controlled trial that compared ventilation with lower versus higher positive end-expiratory pressure (PEEP) in patients with ARDS. The FACTT trial compared liberal fluid therapy versus conservative fluid therapy in patients with ARDS. The SAILS trial compared a placebo with rosuvastatin in patients with ARDS. It is noteworthy that the three RCTs tested different clinical interventions, but all reported negative 60-day mortality results.

### Selection of clinical variables

After the evaluation of data availability and the rate of missing clinical variables in the eICU dataset and the three RCTs (Additional file 1: Figure S2 and Table S1), 21 variables were selected as training variables for the derivation of ARDS phenotypes. These variables were sex; age; temperature; heart rate; respiration rate; systolic blood pressure (SBP); Glasgow Coma Scale score; potassium, sodium, glucose, hematocrit, creatinine, blood urea nitrogen (BUN), bicarbonate, albumin, and total bilirubin levels; platelet count; white blood cell (WBC) count; paO2; paCO2; and pH. For each variable, we extracted the most abnormal value recorded in the 16-h time window surrounding the ARDS diagnosis (8 h before and 8 h after).

### Statistical analysis

Prior to performing the cluster analysis, data cleaning, distribution transformation, extreme value bounding, missing value imputation, and correlation analysis were performed to ensure the integrity of the data. After examining the distribution of the selected variables, data cleaning was performed to filter out erroneous measurements (SMethods in the Additional file). Following data cleaning, we examined the skewness of the selected variables and applied log transformation to variables with a right-skewed distribution (Additional file 1: Table S2). Extreme value bounding was then applied, and normalization was subsequently performed. To address missing values, we excluded patients with 10 or more missing variables and performed multiple imputation with a chained equation (MICE) (Additional file 1: Table S3). Finally, the correlation matrix of 21 clinical variables was evaluated (Additional file 1: Figure S3).

In cluster analysis, ordering points to identify the clustering structure (OPTICS) are applied to determine the appropriate clustering algorithm. K-means clustering (K-means) was used as the clustering model, and gap and gap* statistics were calculated to determine the optimal number of clusters. Consensus clustering (CC) was applied to evaluate the optimal number of clusters and cluster assignments of K-means under resampling (SMethods in Additional file 1). After the optimal phenotypes were derived, the results were visualized with t-distributed stochastic neighbor embedding (t-SNE), line and rank plots.

To evaluate derived clinical phenotypes, we evaluated the clustering model in the eICU validation cohort and the three RCTs. First, data cleaning, normalization, and imputation were performed, and the clinical phenotypes were predicted (SMethods in Additional file). We studied the stability of the clinical characteristics, frequency, and mortality of each phenotype across different cohorts. In the three RCTs, heterogeneity of treatment effect (HTE) was also evaluated by the interaction test. To confirm that the derived clinical phenotypes were not a simple reconstruction of traditional patient risk stratification systems, we studied (1) the conditional distribution of the APACHE score with respect to different phenotypes, (2) an alluvial plot of the distribution of phenotypes with respect to the Berlin classification of ARDS, and (3) the predictive power of the APACHE score and P/F ratio for the clinical phenotypes.

The clinical characteristics of the phenotypes are presented as the counts with percentages, means with standard deviations (SDs) or medians with interquartile ranges (IQRs), as appropriate. Chi-square and Kruskal–Wallis tests were performed where appropriate. Heterogeneity of the treatment effect was tested by the interaction term of the logistic regression for mortality and Poisson regression for ICU-free days (IFD) and ventilator-free days (VFD). Level of significance for test of interaction was adjusted to 0.0167 according to Bonferroni correction. Analyses were performed with Python 3.6.7 (Python Software Foundation) and R version 3.6.3 (R Foundation for Statistical Computing).

## Results

### Patients in the study

For the eICU database, a total of 10,291 patients met the diagnostic criteria for ARDS, and 7545 patients were enrolled in the study. Among the enrolled population, 3875 patients were included in the eICU derivation cohort, and 3670 patients were included in the eICU validation cohort (Additional file 1: Figure S4). The in-hospital mortality rates and severity of illness measured by APACHE IV scores were similar across the two cohorts (Additional file 1: Table S4). A total of 2289 patients (549 from ALVEOLI, 995 from FACTT and 745 from SAILS) from the three RCTs were enrolled in the study. The clinical characteristics of patients from the three RCTs can be found in Additional file 1: Table S5.

### Derivation of three ARDS phenotypes

**Fig. 1:**
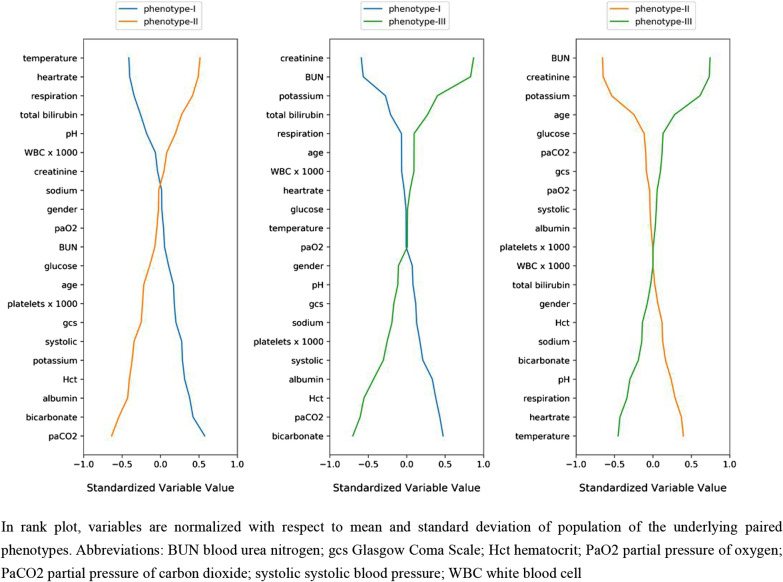
Rank plot of variable mean among paired phenotypes on eICU derivation cohort

Based on OPTICS, gap statistics, cluster consensus and clustering stability, the optimal number of clusters was determined to be 3 (Additional file 1: Figure S5-9). The derived phenotypes were visualized by line and t-SNE plots (Additional file 1: Figure S10-11). The sizes and baseline characteristics of the three phenotypes in the eICU derivation cohort are presented in Table 1 and Additional file 1: Table S6. The three phenotypes had different clinical characteristics and organ failure patterns. We ranked the contribution of each variable to the clinical phenotype in Fig. 1. Phenotype I was associated with fewer abnormal laboratory values and less organ failure. Phenotype II was characterized by a higher WBC count, temperature, heart rate and respiratory rate, a lower SBP and a younger age. Phenotype III was characterized by an older age, elevated serum creatinine and BUN levels, and lower serum bicarbonate levels. Intraphenotype differences in sex, glucose level, sodium level, and partial pressure of oxygen were not significant. The reproducibility of the derived clinical phenotypes was evaluated, and we found that both the phenotype size and clinical characteristics of the validation cohort and the three RCTs were similar to those of the derivation cohort (Additional file 1: Table S7-10). The biomarker IL-6 was assessed in the ALVEOLI trial. Phenotype II patients have higher IL-6 levels (median: 421.5, IQR: 149.5–1447.2) than Phenotype I patients (median: 164.0, IQR: 67.0–398.5) and Phenotype III patients (median: 246.0, IQR: 95.5–939.0) (Table S8).

**Table 1:** Characteristics of the phenotypes on eICU derivation cohort

Characteristic	eICU derivation	phenotype-I	phenotype-II	phenotype-III
No. of patients (%)	3875	1565	1232	1078
Age, mean (SD), years	66.0 (15.5)	67.3 (14.6)	61.2 (16.7)	69.6 (13.9)
Gender—female, No. (%)	1825 (47.1%)	787 (50.3%)	593 (48.1%)	445 (41.3%)
Past history				
Hypertension (%)	2177 (56.7%)	905 (58.4%)	569 (46.6%)	703 (65.6%)
Insulin-dependent diabetes (%)	608 (15.8%)	228 (14.7%)	131 (10.7%)	249 (23.2%)
COPD (%)	975 (25.4%)	506 (32.7%)	223 (18.3%)	246 (22.9%)
CABG (%)	248 (6.5%)	102 (6.6%)	47 (3.8%)	99 (9.2%)
APACHE IV Score, mean (SD)	62.6 (26.1)	52.6 (20.5)	65.4 (27.6)	74.2 (26.3)
Berlin classification*				
Mild (%)	424 (25.0%)	185 (27.2%)	106 (19.5%)	133 (28.4%)
Moderate (%)	743 (43.9%)	345 (50.7%)	217 (39.9%)	181 (38.6%)
Severe (%)	526 (31.1%)	150 (22.1%)	221 (40.6%)	155 (33.0%)
Temperature, mean (SD), °C	37.3 (0.8)	37.0 (0.6)	37.8 (0.9)	37.0 (0.7)
Heart rate, mean (SD), BPM	104.2 (22.7)	97.4 (19.0)	117.5 (22.2)	98.8 (21.3)
Respiratory rate, mean (SD), breaths/min	28.6 (8.3)	26.3 (7.1)	32.6 (9.1)	27.4 (7.1)
Systolic blood pressure, mean (SD), mm Hg	102.2 (21.9)	109.6 (21.5)	96.6 (18.9)	98.3 (23.0)
Glasgow Coma Scale score, mean (SD)	12.3 (3.7)	13.1 (3.2)	11.4 (4.1)	12.1 (3.6)
Potassium, mean (SD), mg/L	4.1 (0.7)	4.1 (0.6)	3.7 (0.5)	4.5 (0.7)
Sodium, mean (SD), mg/L	137.5 (5.7)	137.9 (5.3)	137.9 (6.0)	136.3 (5.7)
Glucose, median (IQR), mg/dL	131.0 (107.0–168.0)	134.0 (110.0–170.0)	125.0 (104.0–153.0)	135.0 (105.0–185.0)
Hematocrit, mean (SD), g/dL	33.8 (7.2)	37.3 (6.5)	32.3 (6.7)	30.6 (6.4)
Creatinine, median (IQR), mg/dL	1.2 (0.8–1.9)	1.0 (0.7–1.3)	1.0 (0.7–1.4)	2.6 (1.8–4.4)
Blood urea nitrogen, median (IQR), mg/dL	24.0 (16.0–39.0)	20.0 (14.0–28.0)	18.0 (12.0–27.0)	50.0 (36.0–67.0)
Bicarbonate, mean (SD), mmol/L	25.3 (6.4)	29.3 (5.8)	23.4 (5.1)	21.5 (5.2)
Platelets, median (IQR), × 109 /L	206.0 (150.0–275.0)	217.0 (171.8–286.2)	196.0 (131.0–264.0)	191.0 (129.0–265.0)
White blood cell count, median (IQR), × 109 /L	11.6 (8.2–16.1)	10.9 (8.1–14.6)	12.4 (8.5–17.5)	12.1 (8.3–17.0)
Albumin, mean (SD), g/dL	2.9 (0.7)	3.3 (0.6)	2.7 (0.7)	2.8 (0.7)
Total Bilirubin, median (IQR), mg/dL	0.6 (0.4–1.0)	0.5 (0.4–0.8)	0.7 (0.5–1.2)	0.7 (0.4–1.2)
PaO2, mean (SD), mm Hg	73.0 (59.0–97.0)	74.0 (60.0–95.0)	71.0 (58.0–96.0)	75.4 (59.6–100.0)
PaCO2, mean (SD), mm Hg	41.0 (34.0–51.0)	51.0 (42.5–65.0)	36.0 (31.0–41.0)	38.0 (31.0–45.0)
pH, mean (SD), unit	7.3 (0.1)	7.3 (0.1)	7.4 (0.1)	7.3 (0.1)
In-hospital days^†^, median (IQR), d	6.0 (3.0–11.0)	5.0 (3.0–9.0)	7.0 (4.0–13.0)	6.0 (3.0–11.0)
In-hospital mortality, No. (%)	572 (14.8%)	117 (7.5%)	219 (17.8%)	236 (21.9%)

eICU, telehealth intensive care unit; APACHE, Acute Physiology and Chronic Health Evaluation; CABG, coronary artery bypass graft; COPD, chronic obstructive pulmonary disease; BPM, beats per minute; SD, standard deviation; IQR, interquartile range; PaO2, partial pressure of oxygen; PaCO2, partial pressure of carbon dioxide; WBC, white blood cell count

*Berlin classification for 1693 patients with valid arterial blood gas (ABG) test in eICU derivation cohort

^†^In-hospital days calculated as the time difference in days between ICU admission and hospital discharge

### Relationship between phenotypes and clinical outcomes

**Fig. 2:**
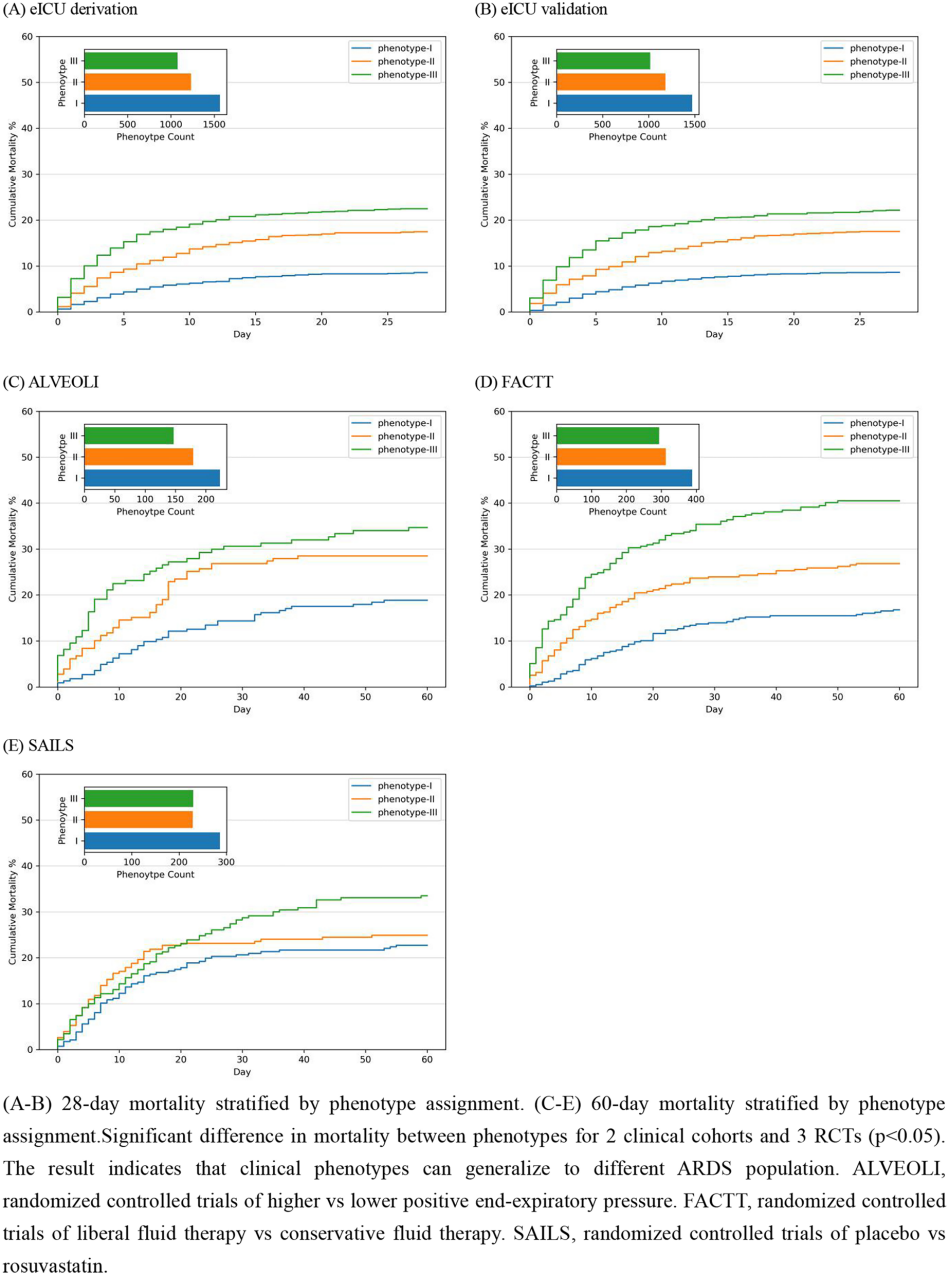
Morality by phenotypes

The three derived phenotypes had distinct clinical outcomes, and those distinctions were consistent in the other four cohorts. All cohorts showed significant differences in mortality by phenotype (*p* < 0.05, Fig. 2). In the eICU derivation and validation cohorts, phenotype I patients had a much lower in-hospital mortality rate (7.5% and 7.4%) than phenotype II (17.8% and 17.1%) and phenotype III (21.9% and 21.7%) patients. In the three RCTs, the mortality rate followed similar patterns, where phenotype I had the lowest 60-day mortality rate (18.8–22.7%) and phenotype III had the highest 60-day mortality rate (33.5–40.5%). The three derived phenotypes demonstrated significant differences in VFD and IFD across the three RCTs (*p* < 0.001, Additional file 1: Table S11). Patients assigned to phenotype I had the most VFD (median: 19–22 days) and IFD (median: 17–19 days), whereas patients assigned to phenotype III had the least VFD (median: 10–17 days) and IFD (median: 7–14 days).

### Heterogeneity of treatment effects

Heterogeneity in treatment effects was detected in two RCTs. In the ALVEOLI trial, significant effects of the interaction between phenotype and PEEP strategy were identified for VFD and IFD (Additional file 1: Figure S12; *p* < 0.01). Patients treated with higher PEEP had fewer VFD and IFD in phenotype I (Table 2). In the FACTT trial, we identified a significant effect of the interaction between phenotype and treatment strategy on 60-day mortality (Additional file 1: Figure S13; *p* = 0.011); mortality among phenotype II patients was 22% with the fluid conservative strategy versus 32% with the fluid liberal strategy, while mortality among phenotype III patients was 45% with the fluid conservative strategy versus 36% with the fluid liberal strategy (Table 3). No strong HTE was observed in the SAILS trial (Additional file 1: Figure S14).

**Table 2:** Differences in response to PEEP strategy by phenotype (ALVEOLI cohort)

PEEP Strategy	phenotype-I		phenotype-II		phenotype-III	
	Low PEEP (*n* = 108)	High PEEP (*n* = 115)	Low PEEP (*n* = 97)	High PEEP (*n* = 82)	Low PEEP (*n* = 68)	High PEEP (*n* = 79)
60-day mortality	14.8%	22.6%	28.9%	28.1%	35.3%	34.2%
Ventilator-free days	18.0	15.5	12.1	12.3	10.7	11.3
ICU-free days	16.1	14.2	9.9	10.8	9.1	10.4

For test of interaction, 60-day mortality of phenotype-I versus II *p* = 0.25, phenotype-I versus III *p* = 0.25, phenotype-II versus III *p* = 0.99; ventilator-free days (VFD) of phenotype-I versus II *p* = 0.002, phenotype-I versus III *p* = 0.001, phenotype-II versus III *p* = 0.67; ICU-free days (IFD) of phenotype-I versus II *p* < 0.001, phenotype-I versus III *p* < 0.001, phenotype-II versus III *p* = 0.47. VFD: number of days during the 28-day period where patients are both alive and free of mechanical ventilation; IFD: number of days during the 28-day period where patients are both alive and free of ICU care; ALVEOLI, randomized controlled trials of higher versus lower PEEP; PEEP, positive end-expiratory pressure; ICU, intensive care unit

**Table 3:** Differences in response to fluid strategy by phenotype (FACTT cohort)

Fluid-management strategy	phenotype-I		phenotype-II		phenotype-III	
	Conservative (*n* = 200)	Liberal (*n* = 188)	Conservative (*n* = 164)	Liberal (*n* = 149)	Conservative (*n* = 137)	Liberal (*n* = 157)
60-day mortality	14.5%	19.2%	22.0%	32.2%	45.3%	36.3%
Ventilator-free days	16.8	14.2	14.3	11.6	10.6	9.4
ICU-free days	14.9	13.0	12.7	10.4	9.4	7.8

For test of interaction, 60-day mortality of phenotype-I versus II *p* = 0.61, phenotype-I versus III *p* = 0.05, phenotype-II versus III *p* = 0.01. Ventilator-free days (VFD) of phenotype-I versus II *p* = 0.39, phenotype-I versus III *p* = 0.27, phenotype-II versus III *p* = 0.08; ICU-free days (IFD) of phenotype-I versus II *p* = 0.15, phenotype-I versus III *p* = 0.29, phenotype-II versus III *p* = 0.83. VFD: number of days during the 28-day period where patients are both alive and free of mechanical ventilation. IFD: number of days during the 28-day period where patients are both alive and free of ICU care. FACTT, randomized controlled trials of liberal fluid therapy versus conservative fluid therapy; ICU, intensive care unit

**Fig. 3:**
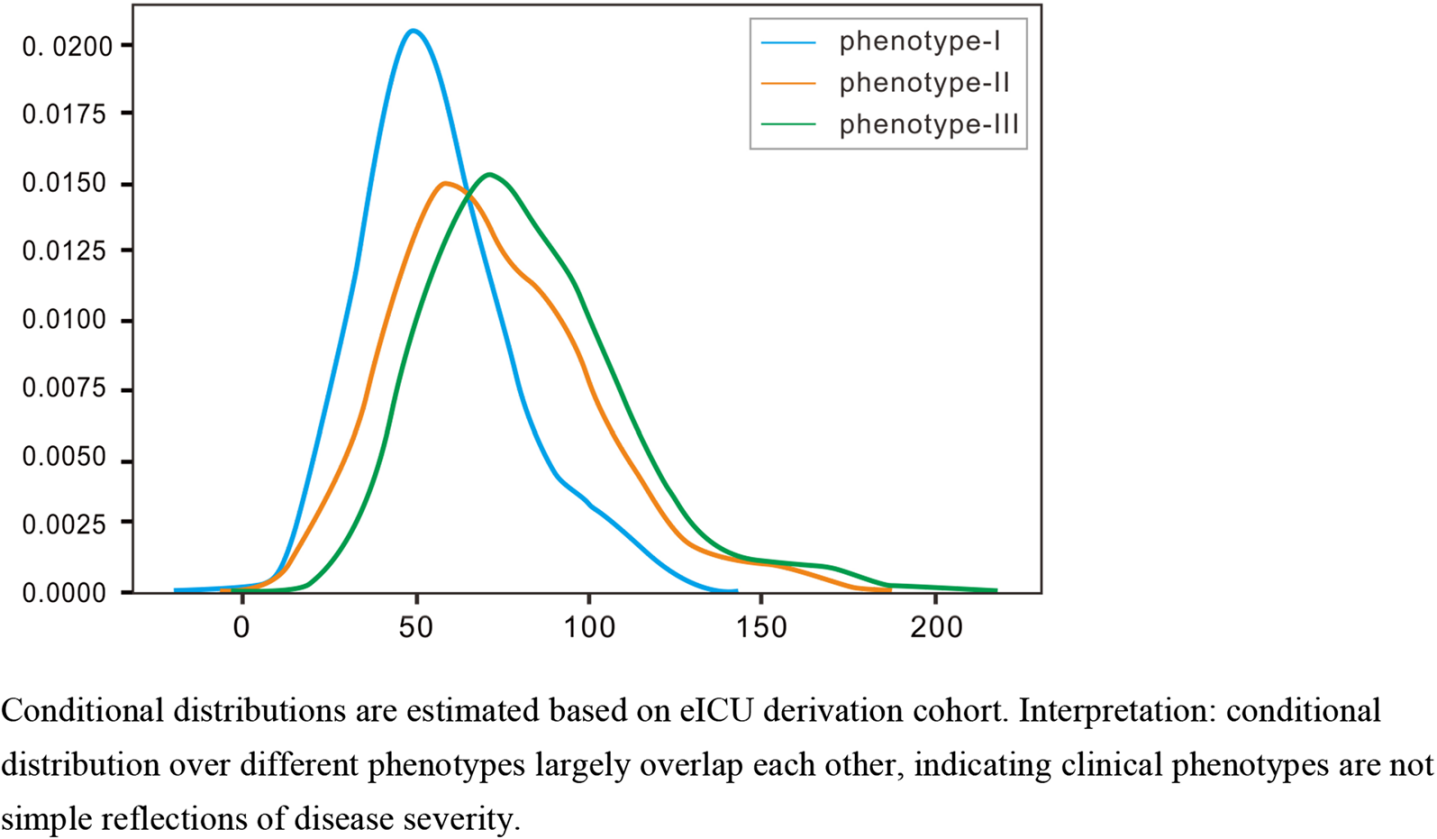
Estimated conditional distribution of APACHE IV score over phenotypes

Finally, we evaluated the relationship between the derived clinical phenotypes and other measures of illness severity. We estimated the distribution of the APACHE score for each phenotype and confirmed that they largely overlapped with each other (Fig. 3). The alluvial plot shows that each Berlin severity level has a significant presence in all clinical phenotypes (Additional file 1: Figure S15). Finally, a logistic regression model was fitted to classify the clinical phenotype using the APACHE score and P/F ratio as predictors. A low ROC AUC confirmed that the derived clinical phenotypes cannot be simply explained by the APACHE score or PaO_2_/FiO_2_ ratio (Additional file 1: Figure S16).

## Discussion

We identified three clinical phenotypes in a retrospective analysis of the eICU database using only routinely available clinical data. The ARDS phenotypes as defined by cluster analysis differed in demographics, patterns of organ failure, laboratory abnormalities, and general illness severity, and the Berlin classification could not fully explain the derived phenotypes. These results were validated across all additional cohorts and trials and were consistent. Furthermore, the three phenotypes were correlated with mortality and other clinical outcomes. We identified a significant interaction between the phenotypes and treatment strategies in both the ALVEOLI and FACTT trials. No strong heterogeneity of treatment effects was observed in the SAILS trial.

To our knowledge, this is the largest study identifying homogeneous phenotypes in ARDS; this study used data from 9834 patients. In addition, three phenotypes were derived from a non-RCT database with more heterogeneous ARDS samples, and only routinely available clinical data were used in the cluster analysis to ensure the generalizability of the model. Indeed, the population characteristics of the ARDS patients in the three RCTs were not exactly the same as those in the eICU database. For example, only 75% of patients in the eICU database had a PaO2/FiO2 ratio < 200. However, in the ALVEOLI and FACTT trials, this number was above 90%. In addition, subjects enrolled in the SAILS trial all had infection-related ARDS [19]. Interestingly, even though the eICU cohorts and RCTs had vastly different ARDS populations, the frequency, clinical characteristics and clinical outcomes of the phenotypes were similar across the five cohorts in our study, which demonstrates the universality of the three phenotypes derived from our clustering model.

To date, progress has been made in several studies with regard to the phenotyping of patients with ARDS. Calfee et al. previously reported two distinct ARDS phenotypes (hyperinflammatory and hypoinflammatory) from a retrospective study of two RCTs, where the patients were clustered by LCA using biomarker and clinical data [13]. Then, a two-phenotype model was reported in the other three RCTs [14, 15, 20] and in one observational cohort [21]. The hyperinflammatory phenotype was characterized by higher plasma concentrations of inflammatory biomarkers and higher mortality than the hypoinflammatory phenotype. Sinha et al. recently reported that hyperinflammatory and hypoinflammatory phenotypes can be accurately predicted without biomarker data using supervised learning [22]. In this study, we generated a three-phenotype model clustered by K-means using clinical data only. Phenotype I was similar to the hypoinflammatory phenotype since both subgroups had fewer abnormal vital and laboratory results, with the lowest mortality risk; it also resembles the previously reported phenotype called ‘rapidly improving ARDS’ or ‘class 3’ [23, 24]. Phenotype II was correlated with inflammation and shock and was associated with a higher mortality rate than phenotype I. Interestingly, phenotype II was similar to the hyperinflammatory phenotype in many ways; specifically, patients in both subgroups had elevated IL-6, elevated heart rate, elevated respiratory rate, and decreased SBP. Phenotype III was correlated with organ dysfunction, older age, and acidosis and had the highest mortality rate. It is possible that organ dysfunction and older age may contribute more to an increased mortality rate than inflammation in these ARDS patients. Compared with the two-phenotype model derived from RCTs, our three-phenotype model may be more comprehensive. The identification of these subsets can help us better understand the heterogeneity of ARDS. Furthermore, the identification of three phenotypes in our study may be useful to propose interventions for patients with a higher risk of mortality (prognostic enrichment). The pathophysiological mechanisms underlying these different phenotypes warrant further exploration.

In our study, three RCTs that had been used to derive the two-phenotype model were selected as validation cohorts to assess the interaction between phenotype and treatment. According to a previous study, the FACTT trial did not show an overall benefit for 60-day mortality based on the type of fluid management received. However, Famous et al. reported that the hyperinflammatory phenotype was associated with different treatment responses to fluid management than the hypoinflammatory phenotype in a two-phenotype model. Our study also revealed the heterogeneity of treatment effects in the FACTT population, and we identified a significant interaction between phenotype and fluid-management strategy in 60-day mortality; specifically, phenotype II patients had lower mortality when randomized to a conservative strategy, while phenotype III patients had higher mortality when randomized to the same strategy. In other words, the conservative fluid therapy strategy reduced the 60-day mortality of phenotype II patients. It is possible that more proinflammatory factors are released into the lung and extrapulmonary organs during severe infection, resulting in microvascular damage [25]. Extravascular fluid tends to accumulate, which can be ameliorated by fluid restriction. Phenotype II derived in our study is similar to the hyperinflammatory phenotype reported by Famous et al., both in clinical characteristic and in response to fluid management. In addition, phenotype III suggests that we should pay attention to fluid management in ARDS patients with renal failure and old age.

In the ALVEOLI trial, significant effects of the interaction between phenotype and PEEP strategy were identified for VFD and IFD in our study. Patients treated with higher PEEP had fewer VFD and IFD in the phenotype I group. It is possible that these patients had no clinically objective positive oxygenation response to a higher PEEP [26]. Similar to the original trial, no treatment benefit of the PEEP strategy with regard to mortality was found for any of the phenotypes. There was no treatment benefit associated with rosuvastatin in any of the three phenotypes in the SAILS trial, and the same result was found in the two-phenotype model. In addition, a retrospective study of SAILS revealed that rosuvastatin may increase the risk of death by raising plasma IL-18 levels [27]. Therefore, it is possible that the negative result may be due to reasons related to the clinical trial design, patient populations and statin choice [11]. Although this study used the same RCT cohorts as the two-phenotype study of Calfee et al. and Sinha et al., the content of our study was not exactly the same. We derived a three-phenotype model in the eICU and validated it in three RCTs rather than through repeated modeling in different RCTs. Furthermore, all reanalyses of three RCTs revealed two ARDS phenotypes, but they differed in the derived variables and clinical characteristics [28]. The variables of the phenotype model in our study were consistent across the five cohorts. Finally, the studies used different analytical approaches. Our study partially overlaps with the previous work of Calfee et al., who used a different methodology, lending credibility to the hypothesis that subphenotypes of ARDS are biologically real.

Our study has some limitations. First, we were not able to obtain the biomarker data for the three RCTs and were not able to perform a head-to-head comparison to hyper-inflammatory or hypo-inflammatory phenotypes. Due to the lack of biomarker data, we cannot determine the biological characteristics of the phenotypes directly and precisely. Second, our study on the three RCTs was a retrospective study, which requires additional validation before treatment benefits can be confirmed. The interaction test only confirms that the two phenotypes responded differently to treatment. Third, missing data were common for some features in the clinical datasets, and we performed multiple imputation before statistical analysis. The missing data could result in some bias in our result. Forth, many data preprocessing decisions had to be made, including variable acquisition time windows, variable selection, and distribution transformation. Changes in decisions may lead to changes in the clustering results and characteristics of phenotypes. Fifth, some patients were excluded from the derivation cohort due to a high level of missing data. However, the empirical studies showed cluster characteristics remained the same when those patients were included.

## Conclusion

Three clinical phenotypes were identified by using routinely available clinical data in a retrospective analysis of the ARDS population in the eICU database, and the derived phenotypes had different clinical characteristics and outcomes. These results were reproduced across all additional cohorts and trials. The analysis provided evidence of a phenotype-specific treatment benefit in the ALVEOLI and FACTT trials. All these findings increase the awareness of the heterogeneity of ARDS and may improve the identification of distinct subsets of patients with ARDS in future randomized controlled trials.

## Supplementary Information

**Additional file 1**. **Figure S1.** Study design. **Figure S2.** Patient selection on the eICU dataset. **Figure S3.** Variable missing heatmap under different extraction time window. **Figure S4.** Heatmap of correlation between clinical variables for phenotyping. **Figure S5.** OPTICS plots for eICU training/validation cohort. **Figure S6.** Gap statistics of K-Means on eICU derivation cohort. **Figure S7.** Consensus k clustering on eICU derivation cohort. **Figure S8.** t-SNE visualization of phenotype assignments by K-Means and consensus clustering. **Figure S9.** Line plot visualization of phenotype characteristics by K-Means and consensus clustering. **Figure S10.** Line plot visualization of phenotype characteristics on 2 clinical cohort. **Figure S11.** t-SNE visualization of phenotype assignments in 2 clinical cohort and 3 RCTs. **Figure S12.** Heterogeneity of treatment effect in ALVEOLI Trail. **Figure S13.** Heterogeneity of treatment effect in FACTT Trail. **Figure S14.** Heterogeneity of treatment effect in SAILS Trail. **Figure S15.** Alluvial plot of relationship between clinical phenotypes and Berlin Classification. **Figure S16.** Predictive power of APACHE score and ARDS severity on derived phenotype. **Table S1.** Availability of selected clinical variables by dataset. **Table S2.** Direction of abnormal values and distribution transformation. **Table S3.** Missing data across cohorts and trials. **Table S4.** Clinical characteristics of eICU derivation/validation cohorts.**Table S5.** Clinical characteristics of 3 RCTs. **Table S6.** Clinical characteristics by phenotype in eICU derivation cohort. **Table S7.** Clinical characteristics by phenotype in eICU validation cohort. **Table S8.** Clinical characteristics by phenotype in ALVEOLI. **Table S9.** Clinical characteristics by phenotype in FACTT. **Table S10.** Clinical characteristics by phenotype in SAILS. **Table S11.** Difference in clinical outcomes between phenotypes in three RCTs.

## Data Availability

Data are available on request.

## Abbreviations

ARDS: Acute respiratory distress syndrome
eICU: Telehealth intensive care unit
RCTs: Randomized controlled trials
PEEP: Positive end-expiratory pressure
PaO2: Partial pressure of arterial oxygen
LCA: Latent class analysis
NHLBI: National Heart, Lung, and Blood Institute
APACHE: Acute Physiology and Chronic Health Evaluation
ICD-9-CM: International Classification of Diseases, Ninth Revision, Clinical Modification
SBP: Systolic blood pressure
BUN: Blood urea nitrogen
WBC: White blood cell
MICE: Multiple imputation with chained equation
OPTICS: Ordering points to identify the clustering structure
CC: Consensus clustering
t-SNE: T-distributed stochastic neighbor embedding
SD: Standard deviations
IQRs: Interquartile ranges
IFD: ICU-free days
VFD: Ventilator-free day
HTE: Heterogeneity of treatment effect
