# Supplementary material for: Identification of distinct clinical phenotypes of acute respiratory distress syndrome with differential responses to treatment
Source: Crit Care. 2021 Aug 30;25:320. doi: 10.1186/s13054-021-03734-y (PMC8404019; doi:10.1186/s13054-021-03734-y)
Supplement: Supplementary file 1 — Additional file 1. Figure S1. Study design. Figure S2. Patient selection on the eICU dataset. Figure S3. Variable missing heatmap under different extraction time window. Figure S4. Heatmap of correlation between clinical variables for phenotyping. Figure S5. OPTICS plots for eICU training/validation cohort. Figure S6. Gap statistics of K-Means on eICU derivation cohort. Figure S7. Consensus k clustering on eICU derivation cohort. Figure S8. t-SNE visualization of phenotype assignments by K-Means and consensus clustering. Figure S9. Line plot visualization of phenotype characteristics by K-Means and consensus clustering. Figure S10. Line plot visualization of phenotype characteristics on 2 clinical cohort. Figure S11. t-SNE visualization of phenotype assignments in 2 clinical cohort and 3 RCTs. Figure S12. Heterogeneity of treatment effect in ALVEOLI Trail. Figure S13. Heterogeneity of treatment effect in FACTT Trail. Figure S14. Heterogeneity of treatment effect in SAILS Trail. Figure S15. Alluvial plot of relationship between clinical phenotypes and Berlin Classification. Figure S16. Predictive power of APACHE score and ARDS severity on derived phenotype. Table S1. Availability of selected clinical variables by dataset. Table S2. Direction of abnormal values and distribution transformation. Table S3. Missing data across cohorts and trials. Table S4. Clinical characteristics of eICU derivation/validation cohorts.Table S5. Clinical characteristics of 3 RCTs. Table S6. Clinical characteristics by phenotype in eICU derivation cohort. Table S7. Clinical characteristics by phenotype in eICU validation cohort. Table S8. Clinical characteristics by phenotype in ALVEOLI. Table S9. Clinical characteristics by phenotype in FACTT. Table S10. Clinical characteristics by phenotype in SAILS. Table S11. Difference in clinical outcomes between phenotypes in three RCTs. [file 13054_2021_3734_MOESM1_ESM.docx]

**Additional file 1**

SMethods

Figure S1. Study design

Figure S2. Patient selection on the eICU dataset

Figure S3. Variable missing heatmap under different extraction time window

Figure S4. Heatmap of correlation between clinical variables for phenotyping

Figure S5. OPTICS plots for eICU training/validation cohort

Figure S6. Gap statistics of K-Means on eICU derivation cohort

Figure S7. Consensus k clustering on eICU derivation cohort

Figure S8. t-SNE visualization of phenotype assignments by K-Means and consensus clustering

Figure S9. Line plot visualization of phenotype characteristics by K-Means and consensus clustering

Figure S10. Line plot visualization of phenotype characteristics on 2 clinical cohort

Figure S11. t-SNE visualization of phenotype assignments in 2 clinical cohort and 3 RCTs

Figure S12. Heterogeneity of treatment effect in ALVEOLI Trail

Figure S13. Heterogeneity of treatment effect in FACTT Trail

Figure S14. Heterogeneity of treatment effect in SAILS Trail

Figure S15. Alluvial plot of relationship between clinical phenotypes and Berlin Classification

Figure S16. Predictive power of APACHE score and ARDS severity on derived phenotype

Table S1. Availability of selected clinical variables by dataset

Table S2. Direction of abnormal values and distribution transformation

Table S3. Missing data across cohorts and trials

Table S4. Clinical characteristics of eICU derivation/validation cohorts

Table S5. Clinical characteristics of 3 RCTs

Table S6. Clinical characteristics by phenotype in eICU derivation cohort

Table S7. Clinical characteristics by phenotype in eICU validation cohort

Table S8. Clinical characteristics by phenotype in ALVEOLI

Table S9. Clinical characteristics by phenotype in FACTT

Table S10. Clinical characteristics by phenotype in SAILS

Table S11. Difference in clinical outcomes between phenotypes in three RCTs

References

SMethods

Step I. Data preprocessing

To ensure data quality and hypothesis of clustering algorithm was satisfied, the following preprocessing steps were performed:

(1) data cleaning

Data cleaning was performed on vital monitoring and lab result data to filter out erroneous measurement and correct unsuccessful default data conversion. We examined the statistics and distribution plot of selected clinical variables and FiO2 which were used in calculation of PaO2/FiO2 ratio (P/F ratio). Erroneous measurement such as pH < 6 were removed from the dataset. Additional data conversion was performed on entries where the default data conversion is unsuccessful. For example, bicarbonate measure recorded as “>45” was converted to 45 and age recorded as “>89” was converted to 89. For calculation of P/F ratio, if multiple arterial blood gas test (ABG test) results were recorded at same timestamp with different FiO2 settings, P/F ratio were marked as missing value since we were unable to map PaO2 measurements to corresponding FiO2 ventilator settings.

(2) distribution transformation

We examined skewness and distribution plot of selected variables and applied log transformation on variables with long trails (e.g. Blood Urea Nitrogen, Creatinine).

(3) extreme value bounding

Due to clustering algorithms are sensitive to outliers, we bounded all measurements to their 0.02 and 0.98 percentile respectively to eliminate erroneous and extreme values.

(4) normalization

Normalization was performed for each variable. Categorical variables are encoded by 0 and 1.

(5) removing observations with missing variables

Large number of missing values indicate high degree of information loss. To ensure observations in eICU derivation cohort contains adequate information for clustering, all patients with 10 or more missing variables were removed.

(6) missing value imputation

We observed missing values in eICU dataset under different variable extraction window.^1^ Due to clustering algorithm did not accept missing value as input, data imputation is required. We assumed: variables are missing at random (MAR). Multiple imputation by chained equations (MICE) was applied to the dataset and all clinical variables were modeled by linear regression.^2,3^ MICE generated 10 independent datasets in the imputation procedure. For visualization, the last imputed dataset was selected to generate plots. For modelling, standard clustering was performed on each of the imputed datasets, and their parameters were pooled to obtain the final model.

(7) correlation analysis

Correlation matrix of selected clinical variables was analyzed.

Step II. Understanding the clustering structure

Data points can group in non-overlapping balls with clear boundaries in between or group in one ball that can be partitioned into multiple subgroup in high dimensional space. To understand the grouping structure of points, we applied a clustering algorithm called ordering points to identify the clustering structure (OPTICS).^4^ OPTICS is a density-based model which can detect clusters in various densities. A reachability plot was produced with OPTICS, which was used to determine if data points are group in non-overlapping balls. If the reachability plot consists multiple segmented curves with points on the tail of one curve significantly higher than most points on another curve, points in the underlying dataset are likely group in non-overlapping balls. If the reachability plot consists one smooth curve, points in the underlying dataset are likely to group in one ball. The reachability plot for both eICU derivation and validation cohort were smooth curves, indicating that points were grouped in one ball and that partition clustering model was preferred.

Step III. Clustering

We used centroid based K-means clustering (K-Means) as the clustering model in this study. To determine the optimal number of cluster k, we examined the Gap statistics and the Gap* statistics. Consensus clustering (CC) is used the cross validation the cluster assignment of K-Means.

To evaluate the clustering result, we considered both theoretical and practical factors including: (1) Goodness of fit (2) Adequate large cluster size (3) Salient difference in clinical characteristics between different phenotypes. Phenotypes were visualized by: (1) t-distributed stochastic neighbor embedding plots (t-SNE)^5^ (2) Line plot of variables normalized by 3 phenotype population mean (3) Rank plot of variables normalized by 2 phenotype population mean.

The optimal number of cluster k is determined by the Gap statistics and the Gap* statistics. Gap statistic compares the total intra-cluster variation for number of cluster k with their expected values under the null hypothesis that distribution has no obvious cluster and is calculated as log of Intra-cluster sum of the pairwise distances.^6^ The difference between Gap statistics and Gap* statistics is that Gap* statistics remove the log function.^7^ In some cases, Gap statistics tend to overestimate the number of clusters but may outperform in the overlapping group cases. In these cases, the optimal number of cluster is determined by the highest Gap statistics value or identifying the first number of cluster k, such that Gap_n_(k) ≥ Gap_n_(k + 1) − s_k+1_. Both Diff value in Gap statistics plot and Gap* statistics plot suggested that optimal number of clusters is 3.

To cross validate the cluster assigned by K-Means, we applied consensus clustering on same data to compare the difference in cluster assignments and phenotype clinical characteristics. Consensus clustering aims to group points into clusters by evaluating degree of confidence or “consensus” of two points are grouped into one cluster under the uncertainty of repeated subsampling.^8^ Pairwise consensus value is defined as “the proportion of time in which two point are grouped in the same cluster” and a high consensus value indicates high goodness of fit. We performed consensus clustering under the ConsensusClusterPlus implementation in R.^9^ We observe a sharp decline in Delta Area plot of CDF from 3 class to 4 class, indicating optimal number of clusters is likely to be 3. Under cluster number=3, cluster consensus for all cluster was above 0.8 and consensus matrix plot suggested that goodness of fit was high.

To further test the stability and robustness of K-Means model, we evaluated the following factors: (1) changes in clinical characteristics of phenotypes between models trained on eICU derivation cohort and eICU validation cohort (2) variance of fitted parameters on multiple imputed datasets by MICE (3) changes in clinical characteristics of each phenotype by altering the clinical variable extraction window from +/-8 hours to +/-12 and +/-24 hours. The clinical characteristics of phenotype generated by K-Means were stable cross all the above tests.

Step IV. Validation of result on RCTs

(A) Predicting phenotype in RCTs

Structural difference between ARDS population in eICU cohort and ARDS population from 3 RCTs were observed. Patients in 3 RCTs are significantly younger (average age: 51, 50, 54 in RCTs vs 66 in eICU) and the population size of mild symptom subjects under Berlin definition is much smaller (mild symptom population size: 4%, 6%, 12% in RCTs vs 25% in eICU). Due to this structural difference, we normalized clinical variables on individual RCT with respect to their population means and standard deviations. To avoid the randomness introduced by the MICE, mean value imputation was performed to ensure the imputation results for all experiments and statistical test results were identical. Trained K-Means algorithm was applied on the imputed dataset to predict phenotypes.

(B) Definition Ventilator Free Days (VFD) and ICU Free Days (IFD)

VFDs are defined as: Number of days during the 28-day period where patients are both alive and free of mechanical ventilation. A patient who is extubated on Day 2 of the study and remains alive and free of the ventilator for the remainder of the 28-day study period, the VFD would be calculated as 26. A patient who is ventilated until death on Day 2 would has a VFD equals to zero.

IFD are defined similarly except for condition changed to patients are both alive and free of ICU care

Step V. Heterogeneity of treatment effect (HTE)

To understand treatment effect on derived phenotypes, we evaluate statistical significance of difference in mortality under different treatment and interaction between treatment and phenotypes.^10^ Heterogeneity of treatment effect is tested by the statistical significance of interaction term (interaction term = treatment flag x phenotype flag) in Logistic Regression (Morality Rate) and Poisson Regression (ICU free time and ventilator free time) between phenotype pairs.

Figure S1. Study design


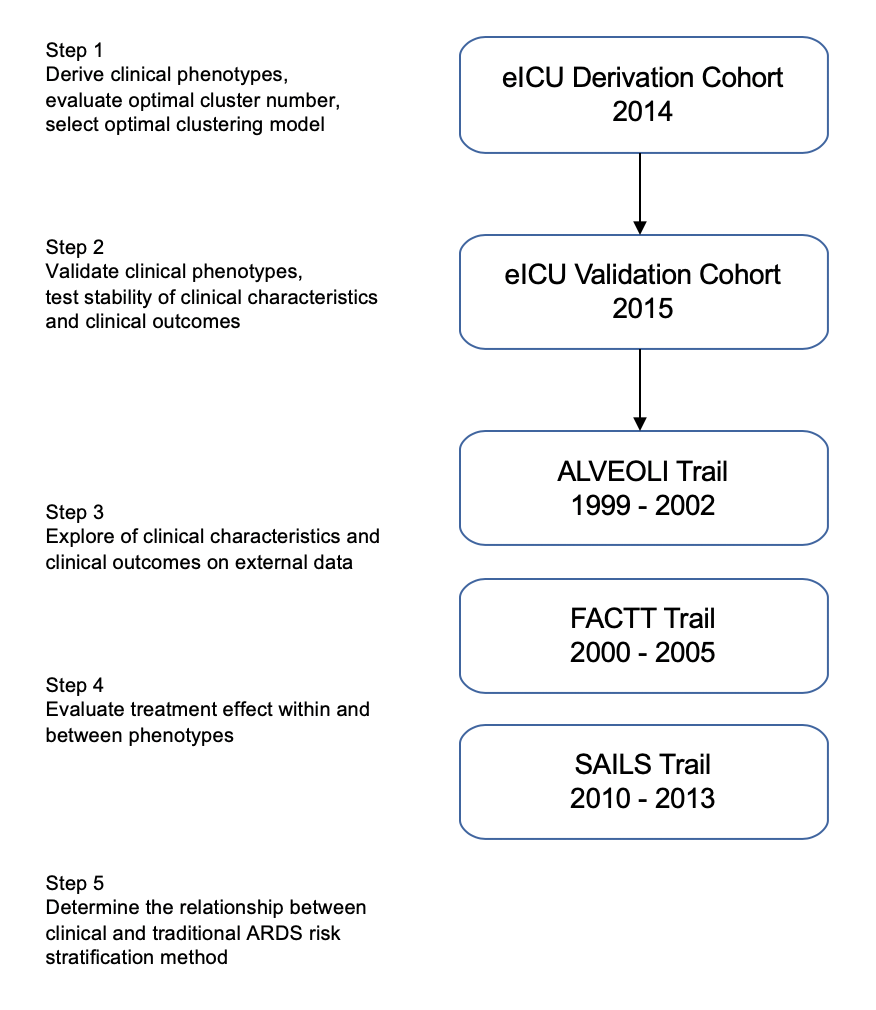


Figure S2. Patient selection on the eICU dataset


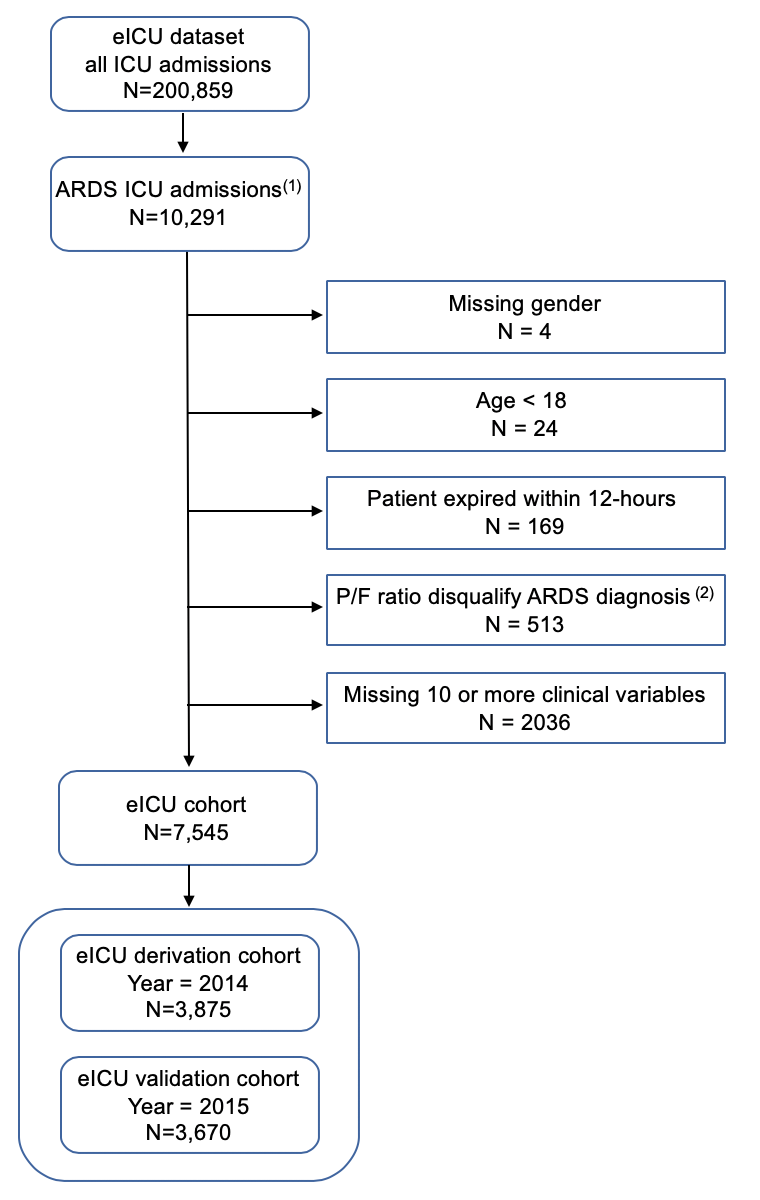


(1) ARDS ICD admissions is defined as ICU admission with APACHE admission diagnosis of 'ARDS-adult respiratory distress syndrome, non-cardiogenic pulmonary edema’ or ICD-9 Diagnosis Code of ‘518.82’

(2) Patients such that the P/F ratio > 300 for all available ABG tests within +/- 24 hours of diagnosis time window were disqualified from ARDS diagnosis and excluded from eICU cohort

(3) All patients with 10 or more missing variables were removed to exclude sample with high degree of information loss

Figure S3. Variable missing heatmap under different extraction time window


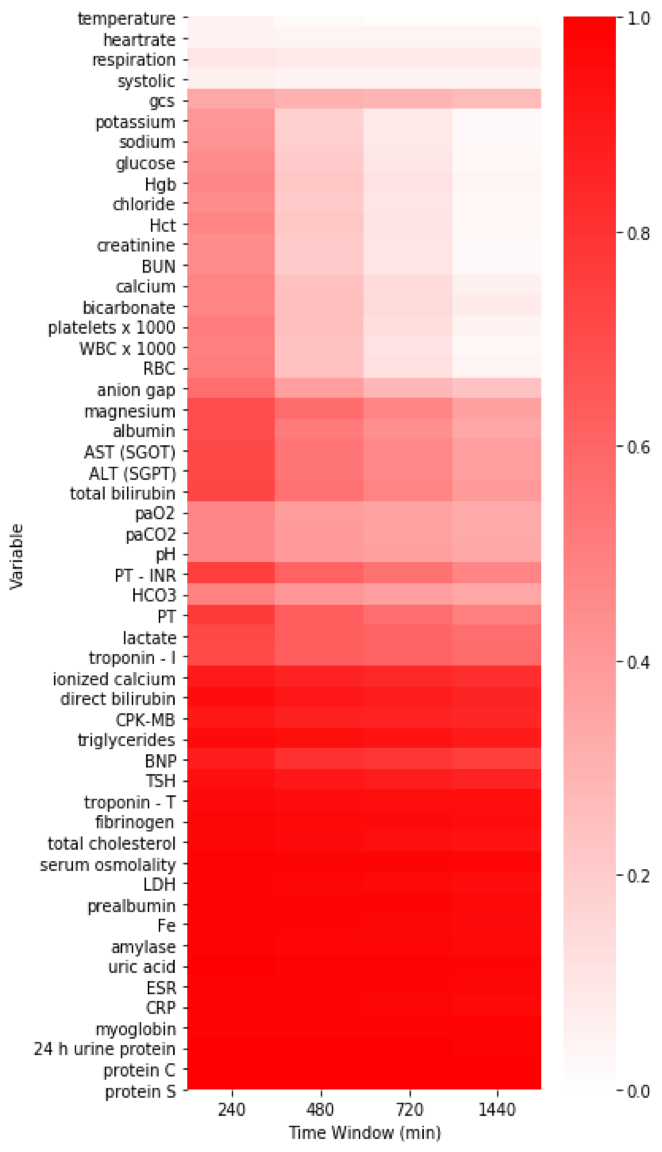


Interpretation: variable missing rate is different across different extraction time-windows. We selected the extraction time-window such that no selected clinical variable has missing rate above 50%

Figure S4. Heatmap of correlation between clinical variables for phenotyping


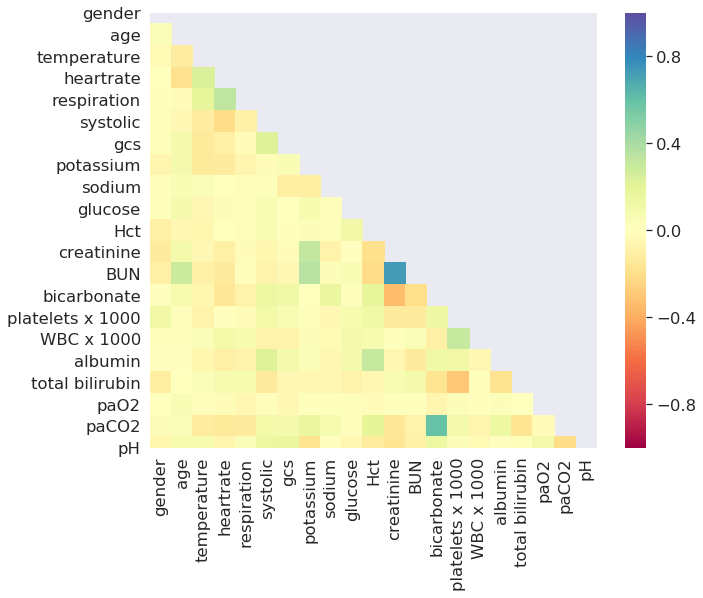


Figure S5. OPTICS plots on eICU derivation / validation cohort

(A) eICU derivation cohort


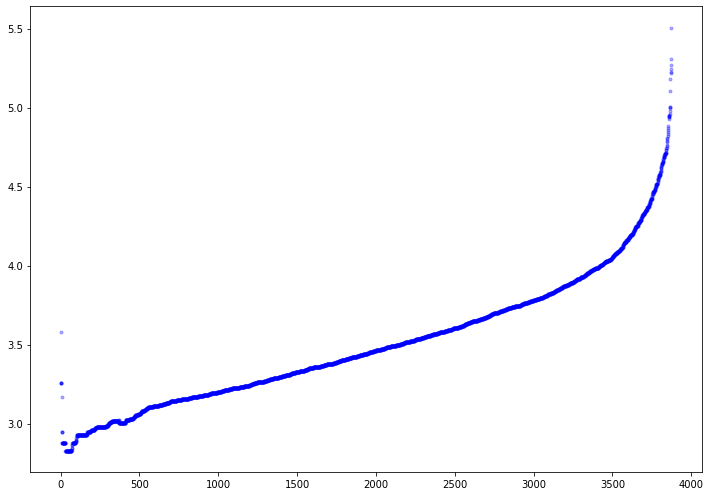


(B) eICU validation cohort


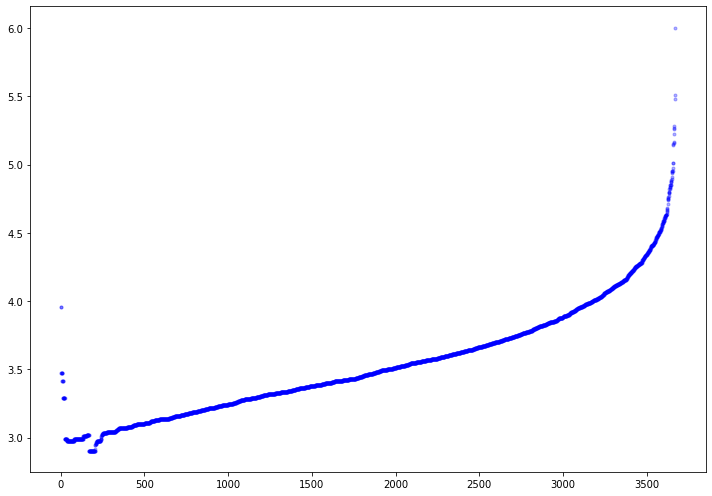


Interpretation: smooth curve in OPTICS plot suggests that data points are likely clustered in a ball. Therefore, we chose centroid based K-means clustering (K-Means) as the clustering model in this study.

Figure S6. Gap statistics and Gap* statistics of K-Means on eICU derivation cohort

| (A)  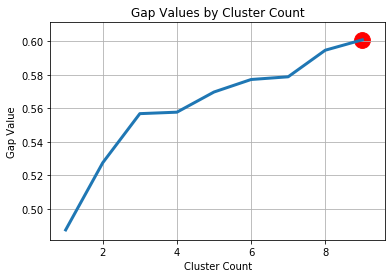 | (B) 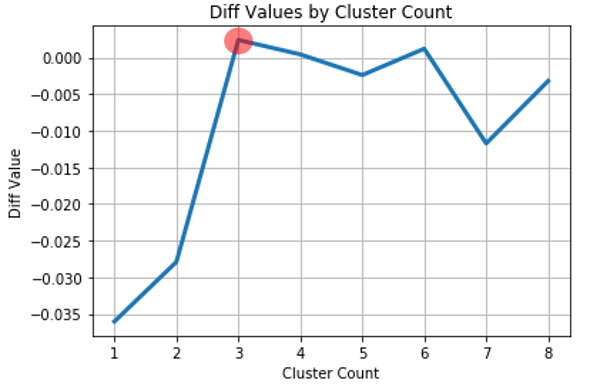 |
| --- | --- |
| (C)  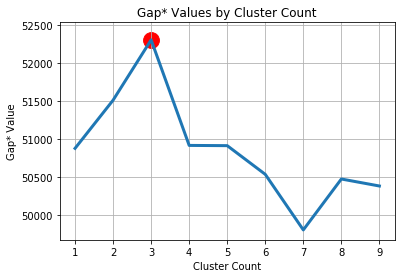 | (D)  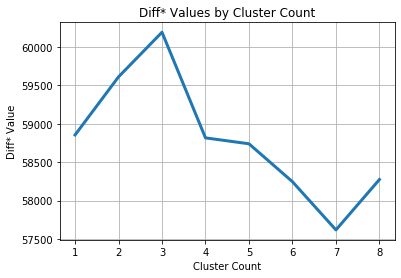 |

(A) Gap statistics of K-Means by cluster count, k

(B) Diff value defined by Gap_n_(k) - Gap_n_(k + 1) + s_k+1_

(C) Gap* statistics of K-Means by cluster count

(D) Diff value defined by Gap*_n_(k) - Gap*_n_(k + 1) + s*_k+1_

Interpretation:

In plot (A), Gap statistics steadily increase over the cluster count, therefore, the optimal number of clusters should be the first k such that Diff value > 0. Plot (B) suggests that optimal number of clusters is 3. In plot (D), Gap* statistics peak at k = 3, suggesting that optimal number of clusters is 3.

Figure S7. Consensus k clustering on eICU derivation cohort

| (A) | (B) |
| --- | --- |
| 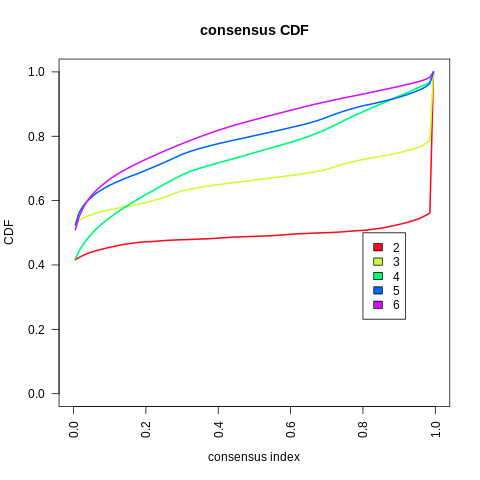 | 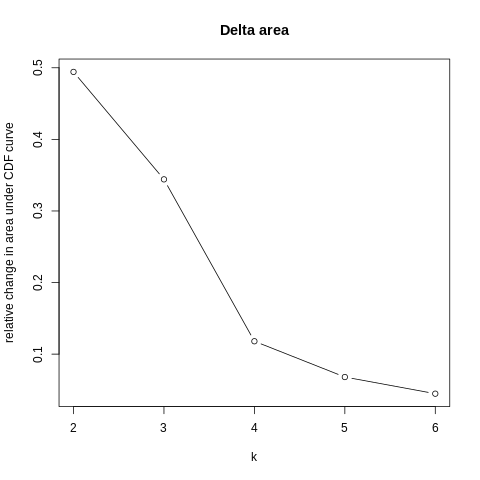 |
| (C) | (D) |
| 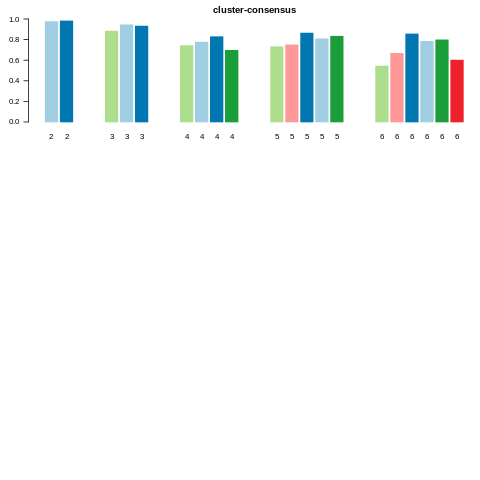 | 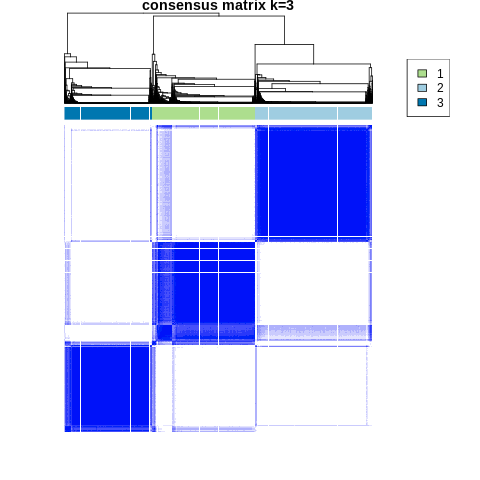 |

(A) Consensus cumulative distribution function (CDF) plot for number of clusters range from 2 to 6

(B) Relative change in area of consensus CDF for number of clusters range from 2 to 6

(C) Cluster consensus plot for number of clusters range from 2 to 6

(D) Visualization of consensus matrix for number of clusters = 3

Interpretation: Plot (B) showed that change in area under CDF curve is around 0.1 from 3 to 4, suggesting that consensus gained from 3 cluster to 4 cluster is limited. Therefore, the optimal number of clusters is 3. Plot (C) suggests that the cluster consensus > 0.8 for all cluster for cluster number =3. Plot (D) is the visualization of consensus matrix for cluster number =3.

Figure S8. t-SNE visualization of phenotype assignments by K-Means and consensus clustering

| No phenotype assignment |  |
| --- | --- |
| 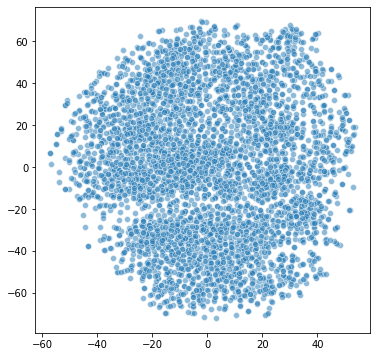 |  |
| Phenotype assigned by K-Means | Phenotype assigned by consensus clustering |
| 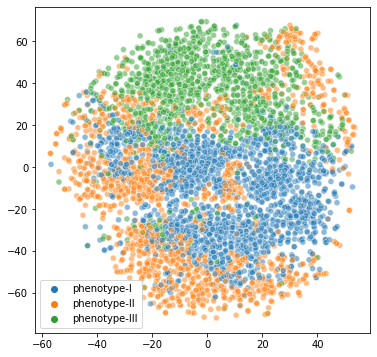 | 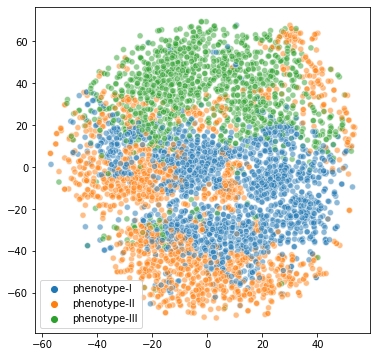 |

Interpretation: No significant difference between phenotypes assigned by K-Means and phenotype assigned by consensus clustering on t-SNE visualization

Figure S9. Line plot visualization of phenotype characteristics by K-Means and consensus clustering

(A) Line plot of phenotype mean of normalized variables by K-Means


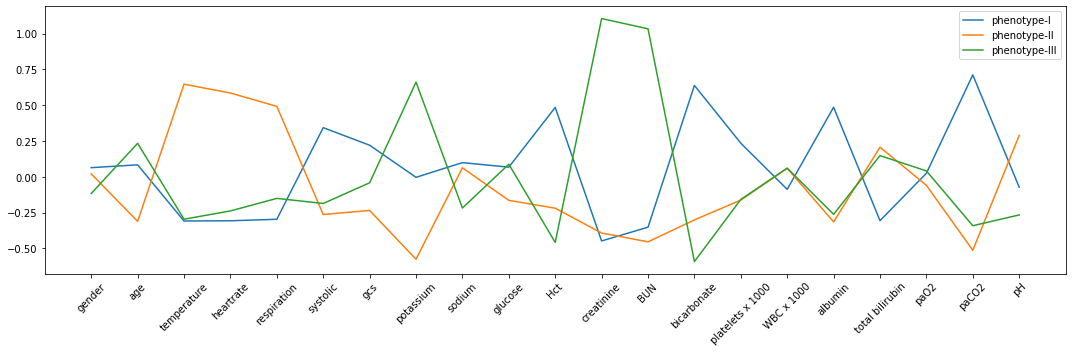


(B) Line plot of phenotype mean of normalized variables by consensus clustering


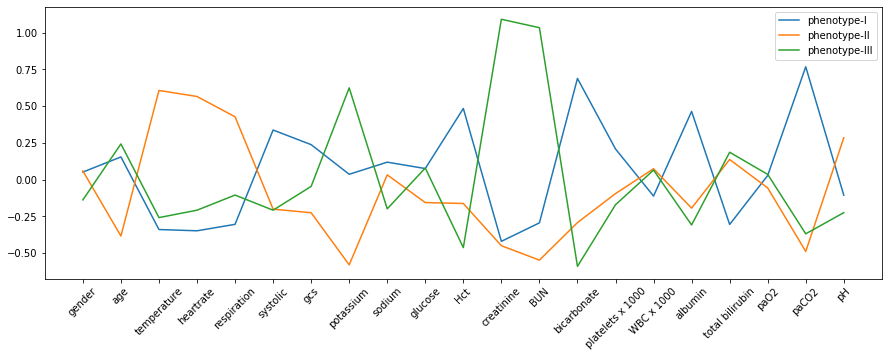


Abbreviations: BUN - blood urea nitrogen; gcs - Glasgow Coma Scale; Hct - hematocrit; PaO2 - partial pressure of oxygen; PaCO2 - partial pressure of carbon dioxide; systolic - systolic blood pressure; WBC x 1000 - white blood cell;

Interpretation: No significant difference in clinical characteristics between phenotypes assigned by K-Means and phenotype assigned by consensus clustering

Figure S10. Line plot of normalized variable means of among all phenotypes

(A) eICU derivation cohort


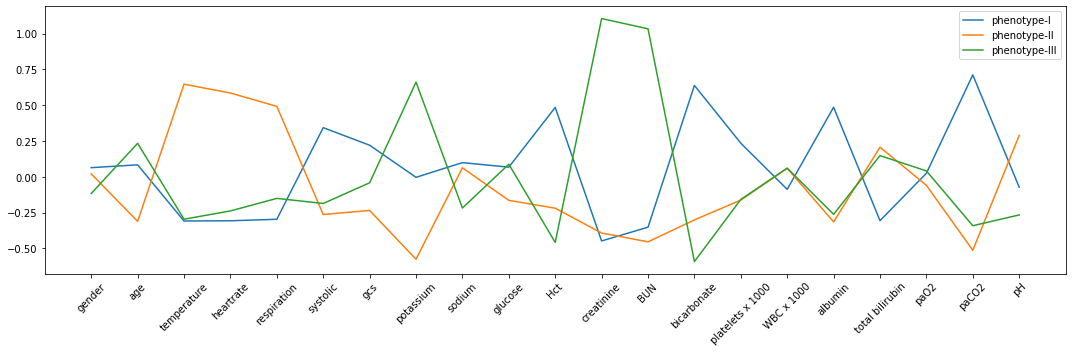


(B) eICU validation cohort


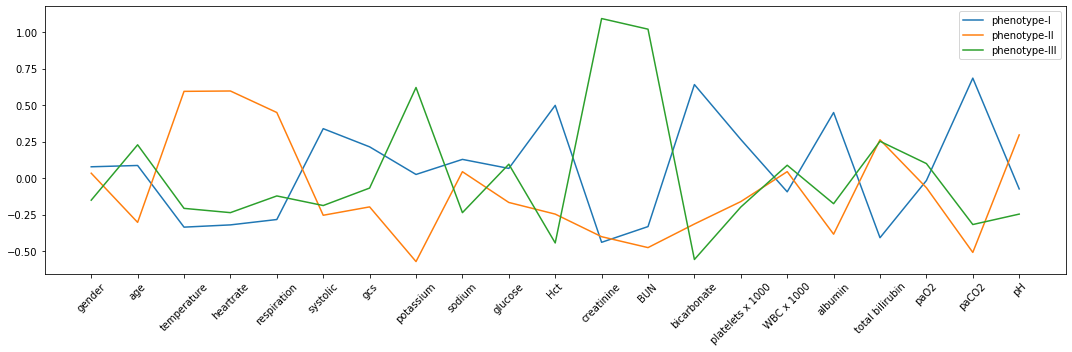


In line plot, variables are normalized with respect to population mean and standard deviation of all 3 phenotypes.

Abbreviations: BUN - blood urea nitrogen; gcs - Glasgow Coma Scale; Hct - hematocrit; PaO2 - partial pressure of oxygen; PaCO2 - partial pressure of carbon dioxide; systolic - systolic blood pressure; WBC x 1000 - white blood cell;

Figure S11. t-SNE plot of phenotype assignments in 2 clinical cohort and 3 RCTs

| (A) eICU derivation | (B) eICU validation |
| --- | --- |
| 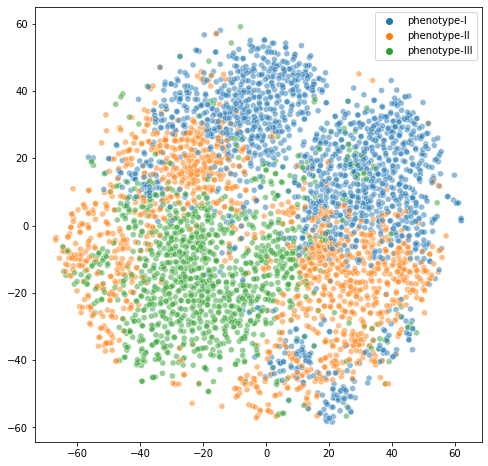 | 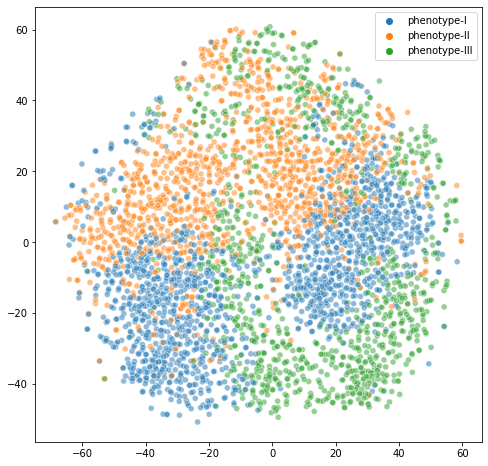 |
| (C) ALVEOLI | (D) FACTT |
| 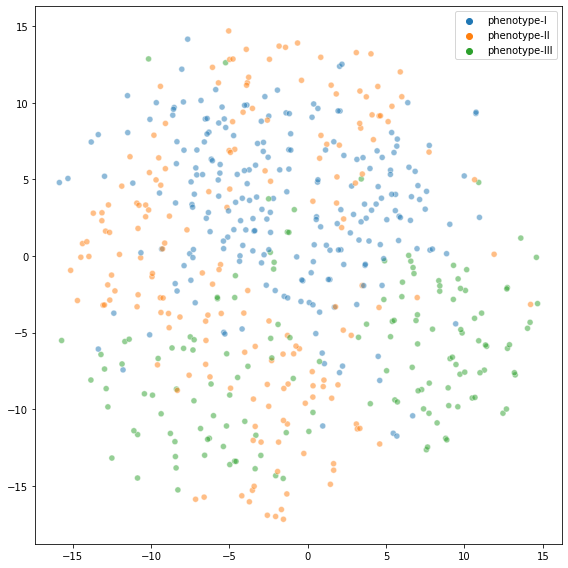 | 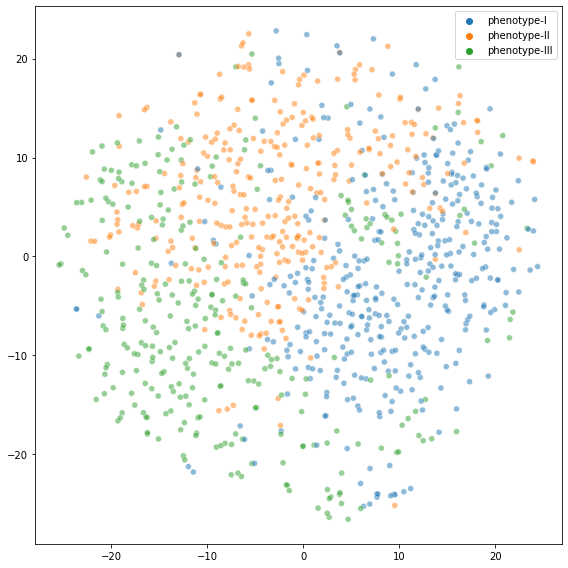 |
| (E) SAILS |  |
| 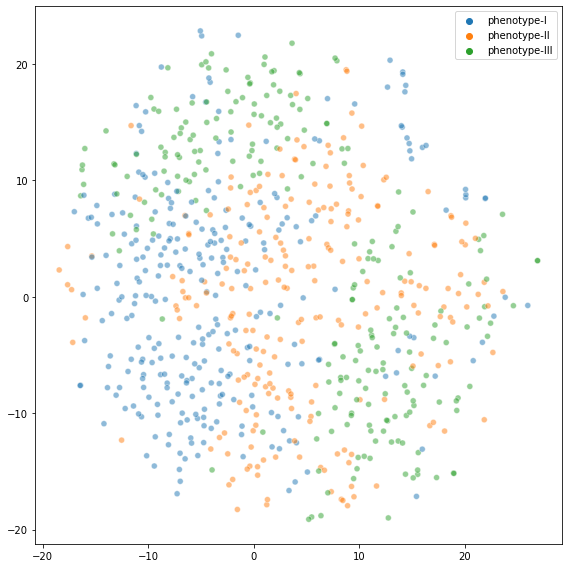 |  |

Interpretation: Embeddings of eICU derivation and eICU validation cohort are different to those of the Figure S8 due to the stochasticity of gradient descent in optimization of t-SNE

Figure S12. Heterogeneity of treatment effect in ALVEOLI Trail

(A)


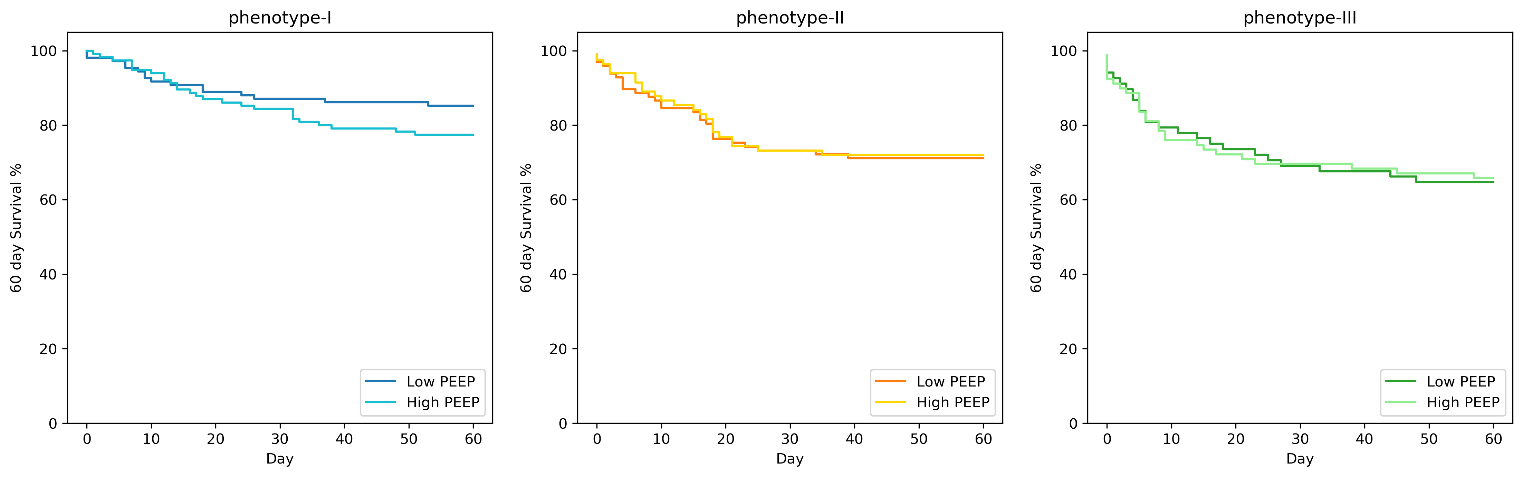


(B)

|  | **phenotype-I** | | **phenotype-II** | | **phenotype-III** | |
| --- | --- | --- | --- | --- | --- | --- |
|  | **Low PEEP (n=108)** | **High PEEP (n=115)** | **Low PEEP (n=97)** | **High PEEP (n=82)** | **Low PEEP (n=68)** | **High PEEP (n=79)** |
| 60-day Mortality | 14.8% | 22.6% | 28.9% | 28.1% | 35.3% | 34.2% |
| Ventilator free days | 18.0 | 15.5 | 12.1 | 12.3 | 10.7 | 11.3 |
| ICU free days | 16.1 | 14.2 | 9.9 | 10.8 | 9.1 | 10.4 |

For test of interaction, Ventilator free days of phenotype-I vs phenotype-II p-value=0.002; VFD of phenotype-I vs phenotype-III p-value=0.001; ICU free days of phenotype-I vs phenotype-II p-value<0.001; IFD of phenotype-I vs phenotype-III p-value<0.001.

(C)

| **Variable** | **Interaction Term** | **Log Odds Ratio** | **Lower Bound 95% CI** | **Upper Bound 95% CI** | **p-value** |
| --- | --- | --- | --- | --- | --- |
| 60-day Mortality | phenotype-I vs phenotype-II | -0.559 | -1.354 | 0.236 | 0.248 |
|  | phenotype-I vs phenotype-III | -0.568 | -1.380 | 0.244 | 0.250 |
|  | phenotype-II vs phenotype-III | -0.009 | -0.800 | 0.782 | 0.985 |
| Ventilator Free Days | phenotype-I vs phenotype-II | 0.171 | 0.082 | 0.260 | 0.002 |
|  | phenotype-I vs phenotype-III | 0.199 | 0.101 | 0.298 | 0.001 |
|  | phenotype-II vs phenotype-III | 0.028 | -0.080 | 0.136 | 0.671 |
| ICU Free Days | phenotype-I vs phenotype-II | 0.209 | 0.113 | 0.304 | 0.000 |
|  | phenotype-I vs phenotype-III | 0.260 | 0.156 | 0.364 | 0.000 |
|  | phenotype-II vs phenotype-III | 0.051 | -0.065 | 0.168 | 0.470 |

(A) 60-day mortality for phenotype stratified by treatment

(B) 60-day mortality, Ventilator-free days, ICU free days of phenotypes stratified by treatment

(C) Test of significance on the interaction term

Figure S13. Heterogeneity of treatment effect in FACTT Trail

(A)


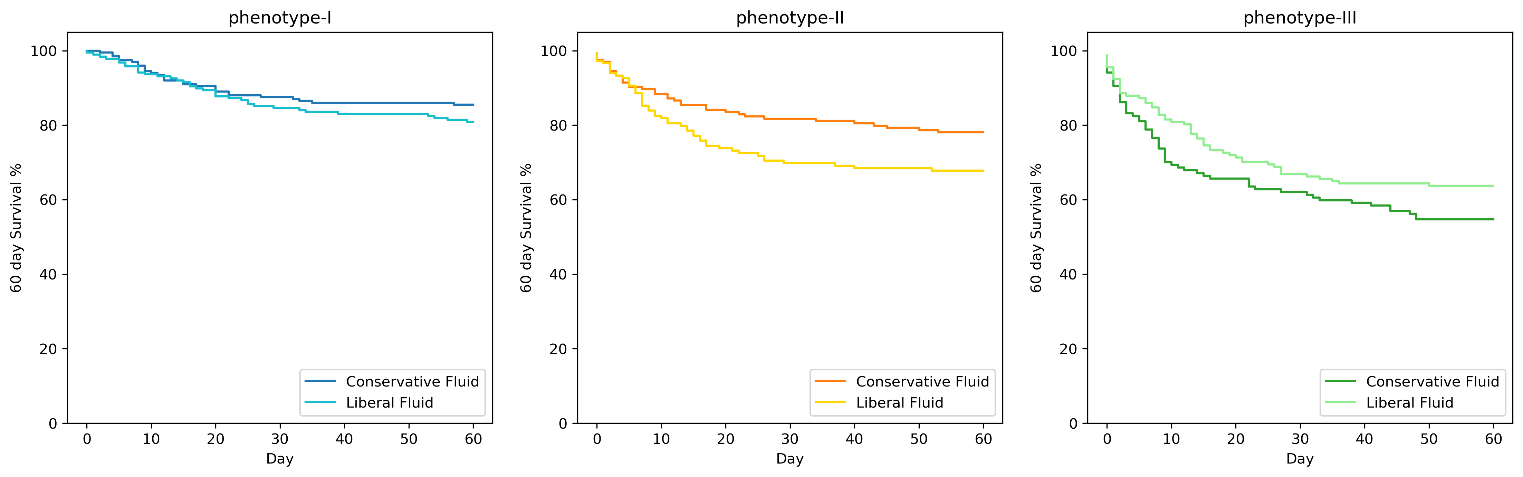


(B)

|  | **phenotype-I** | | **phenotype-II** | | **phenotype-III** | |
| --- | --- | --- | --- | --- | --- | --- |
|  | **conservative (n=200)** | **liberal (n=188)** | **conservative (n=164)** | **liberal (n=149)** | **conservative (n=137)** | **liberal (n=157)** |
| 60-day Mortality | 14.5% | 19.2% | 22.0% | 32.2% | 45.3% | 36.3% |
| Ventilator free days | 16.8 | 14.2 | 14.3 | 11.6 | 10.6 | 9.4 |
| ICU free days | 14.9 | 13.0 | 12.7 | 10.4 | 9.4 | 7.8 |

For test of interaction, 60-day Mortality of phenotype-II vs phenotype-III: p=0.011.

(C)

| **Variable** | **Interaction Term** | **Log Odds Ratio** | **Lower Bound 95% CI** | **Upper Bound 95% CI** | **p-value** |
| --- | --- | --- | --- | --- | --- |
| 60-day Mortality | phenotype-I vs phenotype-II | 0.1906 | -0.427 | 0.808 | 0.612 |
|  | phenotype-I vs phenotype-III | -0.7058 | -1.303 | -0.109 | 0.052 |
|  | phenotype-II vs phenotype-III | -0.8964 | -1.474 | -0.319 | 0.011 |
| Ventilator Free Days | phenotype-I vs phenotype-II | -0.035 | -0.103 | 0.032 | 0.390 |
|  | phenotype-I vs phenotype-III | 0.050 | -0.025 | 0.124 | 0.272 |
|  | phenotype-II vs phenotype-III | 0.085 | 0.005 | 0.165 | 0.082 |
| ICU Free Days | phenotype-I vs phenotype-II | -0.063 | -0.134 | 0.008 | 0.147 |
|  | phenotype-I vs phenotype-III | -0.051 | -0.131 | 0.028 | 0.288 |
|  | phenotype-II vs phenotype-III | 0.011 | -0.074 | 0.097 | 0.827 |

(A) 60-day mortality for phenotype stratified by treatment

(B) 60-day mortality, Ventilator-free days, ICU free days of phenotypes stratified by treatment

(C) Test of significance on the interaction term

Figure S14. Heterogeneity of treatment effect in SAILS Trail

(A)


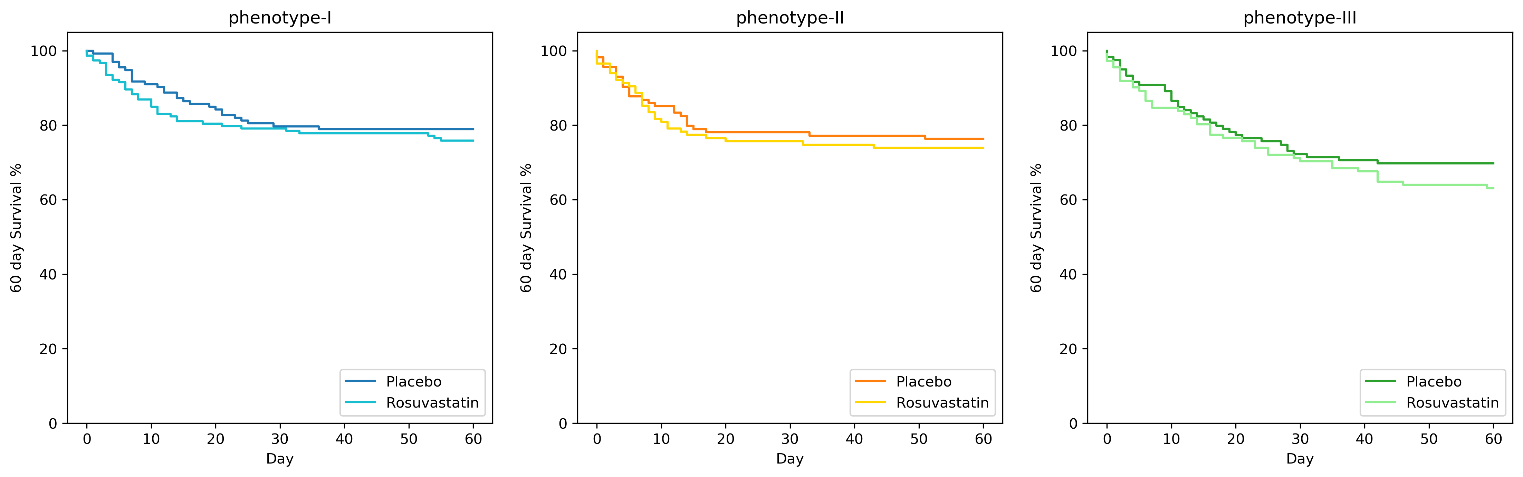


(B)

|  | **phenotype-I** | | **phenotype-II** | | **phenotype-III** | |
| --- | --- | --- | --- | --- | --- | --- |
|  | **placebo (n=133)** | **Rosuvastatin (n=153)** | **placebo (n=114)** | **Rosuvastatin (n=115)** | **placebo (n=119)** | **Rosuvastatin (n=111)** |
| 60-day Mortality | 21.1% | 24.2% | 23.7% | 26.1% | 30.3% | 36.9% |
| Ventilator free days | 17.5 | 16.8 | 14.5 | 15.0 | 12.9 | 13.3 |
| ICU free days | 15.8 | 14.8 | 13.1 | 13.6 | 11.6 | 11.7 |

For test of interaction, ICU Free Days of phenotype-I vs phenotype-II p-value=0.042

(C)

| **Variable** | **Interaction Term** | **Log Odds Ratio** | **Lower Bound 95% CI** | **Upper Bound 95% CI** | **p-value** |
| --- | --- | --- | --- | --- | --- |
| 60-day Mortality | phenotype-I vs phenotype-II | -0.0505 | -0.738 | 0.637 | 0.904 |
|  | phenotype-I vs phenotype-III | 0.1213 | -0.535 | 0.778 | 0.761 |
|  | phenotype-II vs phenotype-III | 0.1718 | -0.511 | 0.854 | 0.679 |
| Ventilator Free Days | phenotype-I vs phenotype-II | 0.072 | -0.002 | 0.145 | 0.110 |
|  | phenotype-I vs phenotype-III | 0.068 | -0.008 | 0.144 | 0.143 |
|  | phenotype-II vs phenotype-III | -0.004 | -0.086 | 0.079 | 0.941 |
| ICU Free Days | phenotype-I vs phenotype-II | 0.096 | 0.018 | 0.173 | 0.042 |
|  | phenotype-I vs phenotype-III | 0.068 | -0.013 | 0.149 | 0.168 |
|  | phenotype-II vs phenotype-III | -0.028 | -0.115 | 0.059 | 0.596 |

(A) 60-day mortality for phenotype stratified by treatment

(B) 60-day mortality, Ventilator-free days, ICU free days of phenotypes stratified by treatment

(C) Test of significance on the interaction term

Figure S15. Alluvial plot of relationship between clinical phenotypes and Berlin Classification

| 1. phenotype-I 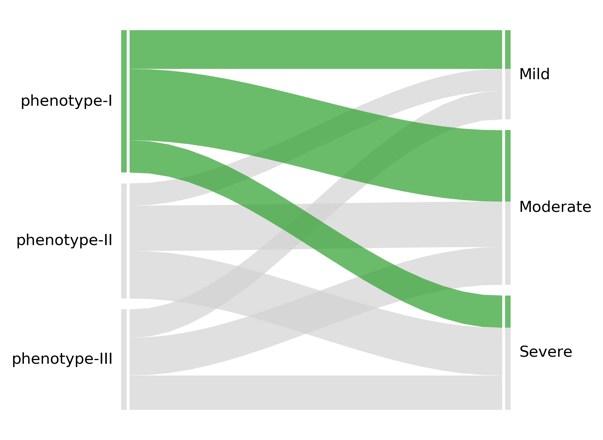 |  |
| --- | --- |
| 1. phenotype-II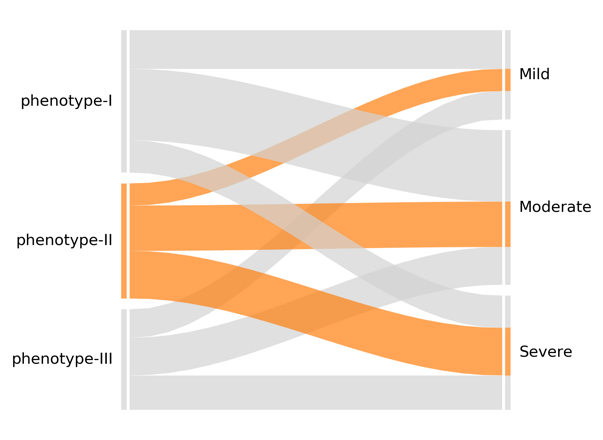 |  |
| 1. phenotype-III 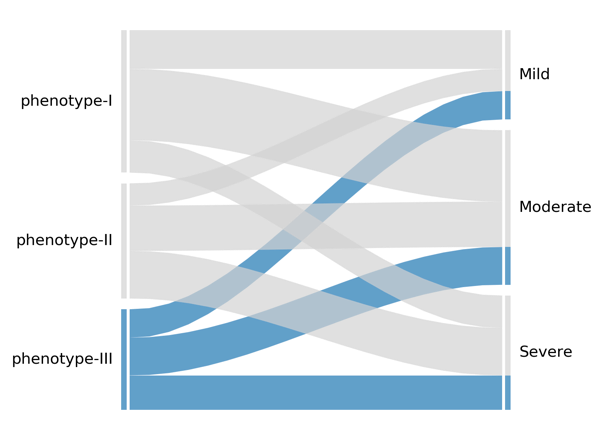 |  |

(A-C) Each phenotype is highlighted separately, and the ribbons connect between phenotype and Berlin classification. The width of ribbon is proportional to the population size. The alluvial plots are based on eICU derivation cohort

Interpretation: patients from one phenotype are classified into all three ARDS severity level according to Berlin definition, indicating clinical phenotypes cannot be fully explained by Berlin definition.

Figure S16. Predictive power of APACHE score and ARDS severity on derived phenotype

| (A) ROC curve of phenotype classification model with APACHE IV score as predictor |
| --- |
| 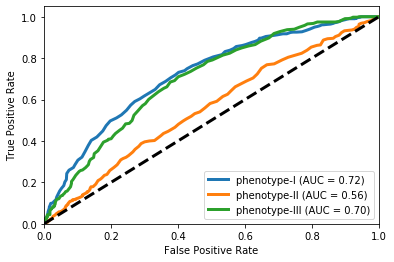 |
| (B) ROC curve of phenotype classification model with P/F ratio as predictor |
| 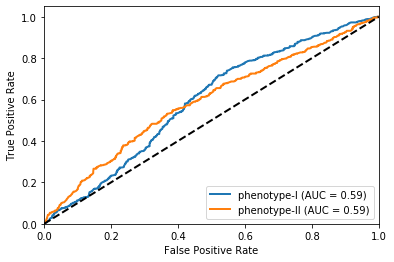 |

(A-B) Classification models are based on eICU derivation cohort

(B) Classification model does not predict any phenotype-III label; therefore, no ROC curve is constructed.

Interpretation: All phenotype classification models which based APACHE score or P/F ratio have AUC ROC < 0.75, indicating clinical phenotypes cannot be fully explained by disease severity or oxygen exchange capacity.

Table S1. Availability of selected clinical variables by dataset

| Variable | eICU derivation | eICU validation | ALVEOLI | FACTT | SAILS |
| --- | --- | --- | --- | --- | --- |
| Age | x | x | x | x | x |
| Gender | x | x | x | x | x |
| Temperature | x | x | x | x | x |
| Heart rate | x | x | x | x | x |
| Respiratory rate | x | x | x | x | x |
| Systolic blood pressure | x | x | x | x | x |
| Glasgow Coma Scale score | x | x | x | x | x |
| Potassium | x | x | x | x | x |
| Sodium | x | x | x | x | x |
| Glucose | x | x | x | x | x |
| Hematocrit | x | x | x | x | x |
| Hemoglobin | x | x |  |  |  |
| Chloride | x | x | x | x |  |
| Creatinine | x | x | x | x | x |
| Blood urea nitrogen | x | x | x | x | x |
| Calcium | x | x |  |  |  |
| Bicarbonate | x | x | x | x | x |
| Platelets | x | x | x | x | x |
| White blood cell count | x | x | x | x | x |
| Red blood cell count | x | x |  |  |  |
| Anion gap | x | x |  |  |  |
| Magnesium | x | x |  |  |  |
| Albumin | x | x | x | x | x |
| AST (SGOT) | x | x |  |  |  |
| ALT (SGPT) | x | x |  |  |  |
| Total Bilirubin | x | x | x | x | x |
| PaO2 | x | x | x | x | x |
| PaCO2 | x | x | x | x | x |
| pH | x | x | x | x | x |
| PT - INR | x | x |  |  |  |
| HCO3 | x | x |  | x |  |
| PT | x | x |  |  |  |
| Lactate | x | x |  |  |  |
| Troponin - I | x | x |  |  |  |

Interpretation: clinical variables are selected such that they are not systematically missing in 3 RCTs

Table S2. Direction of abnormal values and distribution transformation

| Variable | Abnormal value extractor direction | Transformation |
| --- | --- | --- |
| Age | - |  |
| Gender | - |  |
| Temperature | max |  |
| Heart rate | max |  |
| Respiratory rate | max |  |
| Systolic blood pressure | min |  |
| Glasgow Coma Scale score | min |  |
| Potassium | min |  |
| Sodium | min |  |
| Glucose | min | log |
| Hematocrit | min |  |
| Creatinine | max | log |
| Blood urea nitrogen | max | log |
| Bicarbonate | min |  |
| Platelets | min | log |
| White blood cell count | max | log |
| Albumin | min |  |
| Total Bilirubin | max | log |
| PaO2 | min | log |
| PaCO2 | min | log |
| pH | min |  |

Table S3. Missing data across cohorts and trials

| Variable | eICU derivation | eICU validation | ALVEOLI | FACTT | SAILS |
| --- | --- | --- | --- | --- | --- |
| Age | 0 (0.0%) | 0 (0.0%) | 0 (0.0%) | 0 (0.0%) | 0 (0.0%) |
| Gender | 0 (0.0%) | 0 (0.0%) | 0 (0.0%) | 0 (0.0%) | 0 (0.0%) |
| Temperature | 204 (5.26%) | 128 (3.49%) | 1 (0.18%) | 1 (0.1%) | 0 (0.0%) |
| Heart rate | 283 (7.3%) | 257 (7.0%) | 1 (0.18%) | 1 (0.1%) | 0 (0.0%) |
| Respiratory rate | 429 (11.07%) | 374 (10.19%) | 1 (0.18%) | 1 (0.1%) | 4 (0.54%) |
| Systolic blood pressure | 299 (7.72%) | 296 (8.07%) | 1 (0.18%) | 1 (0.1%) | 0 (0.0%) |
| Glasgow Coma Scale score | 1402 (36.18%) | 1021 (27.82%) | 0 (0.0%) | 11 (1.11%) | 8 (1.08%) |
| Potassium | 85 (2.19%) | 114 (3.11%) | 1 (0.18%) | 0 (0.0%) | 1 (0.13%) |
| Sodium | 99 (2.55%) | 100 (2.72%) | 1 (0.18%) | 1 (0.1%) | 0 (0.0%) |
| Glucose | 156 (4.03%) | 167 (4.55%) | 3 (0.55%) | 17 (1.71%) | 1 (0.13%) |
| Hematocrit | 335 (8.65%) | 295 (8.04%) | 1 (0.18%) | 3 (0.3%) | 1 (0.13%) |
| Creatinine | 152 (3.92%) | 147 (4.01%) | 2 (0.36%) | 2 (0.2%) | 1 (0.13%) |
| Blood urea nitrogen | 148 (3.82%) | 147 (4.01%) | 1 (0.18%) | 1 (0.1%) | 5 (0.67%) |
| Bicarbonate | 375 (9.68%) | 339 (9.24%) | 0 (0.0%) | 13 (1.31%) | 4 (0.54%) |
| Platelets | 445 (11.48%) | 394 (10.74%) | 5 (0.91%) | 6 (0.6%) | 0 (0.0%) |
| White blood cell count | 380 (9.81%) | 350 (9.54%) | 4 (0.73%) | 10 (1.01%) | 1 (0.13%) |
| Albumin | 1669 (43.07%) | 1574 (42.89%) | 35 (6.39%) | 214 (21.57%) | 95 (12.77%) |
| Total Bilirubin | 1827 (47.15%) | 1737 (47.33%) | 32 (5.84%) | 260 (26.21%) | 91 (12.23%) |
| PaO2 | 1426 (36.8%) | 1333 (36.32%) | 2 (0.36%) | 7 (0.71%) | 38 (5.11%) |
| PaCO2 | 1459 (37.65%) | 1347 (36.7%) | 2 (0.36%) | 7 (0.71%) | 38 (5.11%) |
| pH | 1483 (38.27%) | 1350 (36.78%) | 2 (0.36%) | 7 (0.71%) | 38 (5.11%) |

Interpretation: no selected clinical variable has missing rate > 50%

Table S4. Clinical characteristics of eICU derivation/validation cohorts

| **Characteristic** | **eICU derivation** | **eICU validation** |
| --- | --- | --- |
| No. of patients | 3875 | 3670 |
| Age, mean (SD), years | 66.0 (15.5) | 65.8 (15.6) |
| Gender - Female, No. (%) | 1825 (47.1%) | 1769 (48.2%) |
| Comorbidities |  |  |
| Hypertension (%) | 2177 (56.7%) | 2125 (58.4%) |
| Insulin dependent diabetes (%) | 608 (15.8%) | 581 (16.0%) |
| COPD (%) | 975 (25.4%) | 1087 (29.9%) |
| CABG (%) | 248 (6.5%) | 266 (7.3%) |
| APACHE IV Score, mean (SD) | 62.6 (26.1) | 63.2 (26.4) |
| Berlin classification |  |  |
| Mild (%) | 424 (25.0%) | 385 (25.5%) |
| Moderate (%) | 743 (43.9%) | 699 (46.3%) |
| Severe (%) | 526 (31.1%) | 427 (28.2%) |
| Temperature, mean (SD), °C | 37.3 (0.8) | 37.2 (1.0) |
| Heart rate, mean (SD), BPM | 104.2 (22.7) | 104.6 (21.9) |
| Respiratory rate, mean (SD), breaths/min | 28.6 (8.3) | 29.3 (8.7) |
| Systolic blood pressure, mean (SD), mm Hg | 102.2 (21.9) | 101.9 (21.6) |
| Glasgow Coma Scale score, mean (SD) | 12.3 (3.7) | 12.3 (3.7) |
| Potassium, mean (SD), mEq/L | 4.1 (0.7) | 4.1 (0.7) |
| Sodium, mean (SD), mEq/L | 137.5 (5.7) | 137.5 (5.6) |
| Glucose, median (IQR), mg/dL | 131.0 (107.0-168.0) | 130.0 (105.0-171.0) |
| Hematocrit, mean (SD), g/dL | 33.8 (7.2) | 33.8 (7.4) |
| Creatinine, median (IQR), mg/dL | 1.2 (0.8-1.9) | 1.2 (0.8-1.9) |
| Blood urea nitrogen, median (IQR), mg/dL | 24.0 (16.0-39.0) | 24.0 (15.0-40.0) |
| Bicarbonate, mean (SD), mmol/L | 25.3 (6.4) | 24.9 (6.5) |
| Platelets, median (IQR), ×10^9^ /L | 206.0 (150.0-275.0) | 205.0 (153.0-275.0) |
| White blood cell count, median (IQR), ×10^9^ /L | 11.6 (8.2-16.1) | 11.8 (8.5-16.4) |
| Albumin, mean (SD), g/dL | 2.9 (0.7) | 3.0 (0.7) |
| Total Bilirubin, median (IQR), mg/dL | 0.6 (0.4-1.0) | 0.6 (0.4-1.0) |
| PaO2, mean (SD), mm Hg | 73.0 (59.0-97.0) | 76.0 (60.8-101.0) |
| PaCO2, mean (SD), mm Hg | 41.0 (34.0-51.0) | 41.0 (34.0-51.0) |
| pH, mean (SD), unit | 7.3 (0.1) | 7.3 (0.1) |
| In-hospital days, median (IQR), d | 6.0 (3.0-11.0) | 6.0 (3.0-10.0) |
| In-hospital mortality, No. (%) | 572 (14.8%) | 531 (14.5%) |

Abbreviations: APACHE - Acute Physiology and Chronic Health Evaluation; CABG - Coronary artery bypass graft; COPD - Chronic obstructive pulmonary disease; BPM - beats per minute; IQR - interquartile range; PaO2 - Partial pressure of oxygen; PaCO2 - Partial pressure of carbon dioxide;

Categorical variables are measured by occurrence and percentage. Normally distributed numerical variables are measured by mean and standard deviation, Non-normally distributed numerical variables are measured by median and interquartile range.

Table S5. Clinical characteristics of 3 RCTs

| **Characteristic** | **ALVEOLI** | **FACTT** | **SAILS** |
| --- | --- | --- | --- |
| No. of patients | 549 | 995 | 745 |
| Age, mean (SD), years | 51.1 (17.2) | 49.7 (16.0) | 54.1 (16.3) |
| Gender - Female, No. (%) | 247 (45.0%) | 462 (46.4%) | 380 (51.0%) |
| APACHE III Score, mean (SD) | - | 94.2 (30.9) | 93.4 (28.2) |
| Berlin classification |  |  |  |
| Mild (%) | 23 (4.2%) | 64 (6.4%) | 85 (12.1%) |
| Moderate (%) | 188 (34.5%) | 386 (38.5%) | 332 (47.3%) |
| Severe (%) | 334 (61.3%) | 533 (55.1%) | 285 (40.6%) |
| Temperature, mean (SD), °C | 38.4 (0.9) | 38.4 (1.0) | 38.1 (1.0) |
| Heart rate, mean (SD), BPM | 125.1 (24.1) | 124.5 (21.8) | 118.4 (22.7) |
| Respiratory rate, mean (SD), breaths/min | 32.7 (10.2) | 34.8 (10.1) | 32.9 (8.5) |
| Systolic blood pressure, mean (SD), mm Hg | 88.2 (16.5) | 88.2 (16.4) | 85.4 (15.6) |
| Glasgow Coma Scale score, mean (SD) | 9.4 (4.3) | 8.0 (4.0) | 7.9 (3.4) |
| Potassium, mean (SD), mEq/L | 3.8 (0.6) | 3.8 (0.6) | 3.8 (0.6) |
| Sodium, mean (SD), mEq/L | 136.9 (5.1) | 137.4 (5.8) | 137.8 (5.4) |
| Glucose, median (IQR), mg/dL | 118.0 (96.0-150.0) | 112.0 (92.0-140.0) | 115.0 (97.0-143.0) |
| Hematocrit, mean (SD), g/dL | 29.9 (5.7) | 29.7 (6.5) | 29.9 (6.1) |
| Creatinine, median (IQR), mg/dL | 1.1 (0.8-1.9) | 1.1 (0.8-1.6) | 1.1 (0.8-1.8) |
| Blood urea nitrogen, median (IQR), mg/dL | 21.0 (13.0-36.0) | 18.0 (12.0-30.8) | 21.0 (13.0-37.0) |
| Bicarbonate, mean (SD), mmol/L | 21.7 (5.5) | 21.2 (5.6) | 21.8 (5.5) |
| Platelets, median (IQR), ×10^9^ /L | 154.0 (82.8-237.5) | 178.0 (102.0-256.2) | 170.0 (101.0-249.0) |
| White blood cell count, median (IQR), ×10^9^ /L | 13.0 (8.7-18.2) | 12.5 (7.6-18.3) | 13.9 (8.6-20.0) |
| Albumin, mean (SD), g/dL | 2.1 (0.6) | 2.2 (0.7) | 2.2 (0.6) |
| Total Bilirubin, median (IQR), mg/dL | 0.8 (0.5-1.5) | 0.8 (0.5-1.6) | 0.8 (0.5-1.4) |
| PaO2, mean (SD), mm Hg | 62.0 (54.0-72.0) | 64.0 (55.0-76.0) | 70.0 (60.2-83.8) |
| PaCO2, mean (SD), mm Hg | 34.0 (29.0-38.0) | 35.0 (30.0-41.0) | 36.0 (31.0-42.0) |
| pH, mean (SD), unit | 7.3 (0.1) | 7.3 (0.1) | 7.3 (0.1) |
| 60 day mortality, No. (%) | 144 (26.2%) | 268 (26.9%) | 199 (26.7%) |
| ICU free days, median (IQR), d | 15.0 (0.0-21.0) | 14.0 (0.0-21.0) | 17.0 (0.0-23.0) |
| Ventilator free days, median (IQR), d | 17.0 (0.0-23.0) | 17.0 (0.0-22.0) | 19.0 (3.0-24.0) |

Abbreviations: APACHE - Acute Physiology and Chronic Health Evaluation; CABG - Coronary artery bypass graft; COPD - Chronic obstructive pulmonary disease; BPM - beats per minute; IQR - interquartile range; PaO2 - Partial pressure of oxygen; PaCO2 - Partial pressure of carbon dioxide;

Categorical variables are measured by occurrence and percentage. Normally distributed numerical variables are measured by mean and standard deviation, Non-normally distributed numerical variables are measured by median and interquartile range.

Table S6. Clinical characteristics by phenotype in eICU derivation cohort

| **Characteristic** | **phenotype-I** | **phenotype-II** | **phenotype-III** |
| --- | --- | --- | --- |
| No. of patients | 1565 | 1232 | 1078 |
| Age, mean (SD), years | 67.3 (14.6) | 61.2 (16.7) | 69.6 (13.9) |
| Gender - Female, No. (%) | 787 (50.3%) | 593 (48.1%) | 445 (41.3%) |
| Comorbidities |  |  |  |
| Hypertension (%) | 905 (58.4%) | 569 (46.6%) | 703 (65.6%) |
| Insulin dependent diabetes (%) | 228 (14.7%) | 131 (10.7%) | 249 (23.2%) |
| COPD (%) | 506 (32.7%) | 223 (18.3%) | 246 (22.9%) |
| CABG (%) | 102 (6.6%) | 47 (3.8%) | 99 (9.2%) |
| APACHE IV Score, mean (SD) | 52.6 (20.5) | 65.4 (27.6) | 74.2 (26.3) |
| Berlin classification |  |  |  |
| Mild (%) | 185 (27.2%) | 106 (19.5%) | 133 (28.4%) |
| Moderate (%) | 345 (50.7%) | 217 (39.9%) | 181 (38.6%) |
| Severe (%) | 150 (22.1%) | 221 (40.6%) | 155 (33.0%) |
| Temperature, mean (SD), °C | 37.0 (0.6) | 37.8 (0.9) | 37.0 (0.7) |
| Heart rate, mean (SD), BPM | 97.4 (19.0) | 117.5 (22.2) | 98.8 (21.3) |
| Respiratory rate, mean (SD), breaths/min | 26.3 (7.1) | 32.6 (9.1) | 27.4 (7.1) |
| Systolic blood pressure, mean (SD), mm Hg | 109.6 (21.5) | 96.6 (18.9) | 98.3 (23.0) |
| Glasgow Coma Scale score, mean (SD) | 13.1 (3.2) | 11.4 (4.1) | 12.1 (3.6) |
| Potassium, mean (SD), mEq/L | 4.1 (0.6) | 3.7 (0.5) | 4.5 (0.7) |
| Sodium, mean (SD), mEq/L | 137.9 (5.3) | 137.9 (6.0) | 136.3 (5.7) |
| Glucose, median (IQR), mg/dL | 134.0 (110.0-170.0) | 125.0 (104.0-153.0) | 135.0 (105.0-185.0) |
| Hematocrit, mean (SD), g/dL | 37.3 (6.5) | 32.3 (6.7) | 30.6 (6.4) |
| Creatinine, median (IQR), mg/dL | 1.0 (0.7-1.3) | 1.0 (0.7-1.4) | 2.6 (1.8-4.4) |
| Blood urea nitrogen, median (IQR), mg/dL | 20.0 (14.0-28.0) | 18.0 (12.0-27.0) | 50.0 (36.0-67.0) |
| Bicarbonate, mean (SD), mmol/L | 29.3 (5.8) | 23.4 (5.1) | 21.5 (5.2) |
| Platelets, median (IQR), ×10^9^ /L | 217.0 (171.8-286.2) | 196.0 (131.0-264.0) | 191.0 (129.0-265.0) |
| White blood cell count, median (IQR), ×10^9^ /L | 10.9 (8.1-14.6) | 12.4 (8.5-17.5) | 12.1 (8.3-17.0) |
| Albumin, mean (SD), g/dL | 3.3 (0.6) | 2.7 (0.7) | 2.8 (0.7) |
| Total Bilirubin, median (IQR), mg/dL | 0.5 (0.4-0.8) | 0.7 (0.5-1.2) | 0.7 (0.4-1.2) |
| PaO2, mean (SD), mm Hg | 74.0 (60.0-95.0) | 71.0 (58.0-96.0) | 75.4 (59.6-100.0) |
| PaCO2, mean (SD), mm Hg | 51.0 (42.5-65.0) | 36.0 (31.0-41.0) | 38.0 (31.0-45.0) |
| pH, mean (SD), unit | 7.3 (0.1) | 7.4 (0.1) | 7.3 (0.1) |
| In-hospital days, median (IQR), d | 5.0 (3.0-9.0) | 7.0 (4.0-13.0) | 6.0 (3.0-11.0) |
| In-hospital mortality, No. (%) | 117 (7.5%) | 219 (17.8%) | 236 (21.9%) |

Abbreviations: APACHE - Acute Physiology and Chronic Health Evaluation; CABG - Coronary artery bypass graft; COPD - Chronic obstructive pulmonary disease; BPM - beats per minute; IQR - interquartile range; PaO2 - Partial pressure of oxygen; PaCO2 - Partial pressure of carbon dioxide;

Categorical variables are measured by occurrence and percentage. Normally distributed numerical variables are measured by mean and standard deviation, Non-normally distributed numerical variables are measured by median and interquartile range.

Table S7. Clinical characteristics by phenotype in eICU validation cohort

| **Characteristic** | **phenotype-I** | **phenotype-II** | **phenotype-III** |
| --- | --- | --- | --- |
| No. of patients | 1471 | 1183 | 1016 |
| Age, mean (SD), years | 67.2 (14.7) | 61.1 (16.9) | 69.4 (13.8) |
| Gender - Female, No. (%) | 766 (52.1%) | 590 (49.9%) | 413 (40.6%) |
| Comorbidities |  |  |  |
| Hypertension (%) | 893 (61.2%) | 564 (48.2%) | 668 (66.3%) |
| Insulin dependent diabetes (%) | 226 (15.5%) | 125 (10.7%) | 230 (22.8%) |
| COPD (%) | 594 (40.7%) | 254 (21.7%) | 239 (23.7%) |
| CABG (%) | 100 (6.9%) | 60 (5.1%) | 106 (10.5%) |
| APACHE IV Score, mean (SD) | 53.0 (20.8) | 65.6 (27.4) | 74.9 (27.0) |
| Berlin classification |  |  |  |
| Mild (%) | 175 (30.0%) | 99 (19.9%) | 111 (25.9%) |
| Moderate (%) | 294 (50.3%) | 224 (45.0%) | 181 (42.2%) |
| Severe (%) | 115 (19.7%) | 175 (35.1%) | 137 (31.9%) |
| Temperature, mean (SD), °C | 36.9 (1.1) | 37.7 (0.9) | 37.0 (0.7) |
| Heart rate, mean (SD), BPM | 97.6 (18.6) | 117.5 (21.2) | 99.5 (20.4) |
| Respiratory rate, mean (SD), breaths/min | 27.0 (7.5) | 33.0 (9.5) | 28.3 (7.7) |
| Systolic blood pressure, mean (SD), mm Hg | 109.1 (20.6) | 96.4 (19.3) | 97.9 (22.6) |
| Glasgow Coma Scale score, mean (SD) | 13.1 (3.1) | 11.6 (4.0) | 12.0 (3.8) |
| Potassium, mean (SD), mEq/L | 4.2 (0.6) | 3.7 (0.6) | 4.6 (0.7) |
| Sodium, mean (SD), mEq/L | 138.1 (5.0) | 137.7 (5.7) | 136.3 (6.2) |
| Glucose, median (IQR), mg/dL | 133.0 (109.0-172.0) | 125.0 (101.0-155.0) | 134.5 (105.0-184.2) |
| Hematocrit, mean (SD), g/dL | 37.4 (6.8) | 32.0 (6.8) | 30.6 (6.6) |
| Creatinine, median (IQR), mg/dL | 0.9 (0.7-1.3) | 1.0 (0.7-1.3) | 2.6 (1.8-4.4) |
| Blood urea nitrogen, median (IQR), mg/dL | 20.0 (14.0-28.0) | 18.0 (12.0-27.0) | 50.0 (36.0-67.0) |
| Bicarbonate, mean (SD), mmol/L | 29.0 (6.0) | 22.8 (5.1) | 21.3 (5.3) |
| Platelets, median (IQR), ×10^9^ /L | 223.0 (173.0-295.0) | 192.0 (134.0-267.5) | 186.0 (134.0-248.0) |
| White blood cell count, median (IQR), ×10^9^ /L | 10.9 (8.2-14.6) | 12.6 (8.8-17.5) | 12.6 (8.6-17.8) |
| Albumin, mean (SD), g/dL | 3.3 (0.6) | 2.7 (0.7) | 2.9 (0.7) |
| Total Bilirubin, median (IQR), mg/dL | 0.5 (0.3-0.7) | 0.7 (0.5-1.2) | 0.7 (0.4-1.3) |
| PaO2, mean (SD), mm Hg | 75.0 (62.0-96.6) | 73.0 (58.4-100.0) | 81.0 (62.0-110.0) |
| PaCO2, mean (SD), mm Hg | 51.0 (42.4-66.0) | 36.0 (31.0-41.8) | 38.0 (32.0-45.1) |
| pH, mean (SD), unit | 7.3 (0.1) | 7.4 (0.1) | 7.3 (0.1) |
| In-hospital days, median (IQR), d | 5.0 (3.0-9.0) | 6.0 (3.0-12.0) | 6.0 (3.0-11.0) |
| In-hospital mortality, No. (%) | 109 (7.4%) | 202 (17.1%) | 220 (21.7%) |

Abbreviations: APACHE - Acute Physiology and Chronic Health Evaluation; CABG - Coronary artery bypass graft; COPD - Chronic obstructive pulmonary disease; BPM - beats per minute; IQR - interquartile range; PaO2 - Partial pressure of oxygen; PaCO2 - Partial pressure of carbon dioxide;

Categorical variables are measured by occurrence and percentage. Normally distributed numerical variables are measured by mean and standard deviation, Non-normally distributed numerical variables are measured by median and interquartile range.

Table S8. Clinical characteristics by phenotype in ALVEOLI

| **Characteristic** | **phenotype-I** | **phenotype-II** | **phenotype-III** |
| --- | --- | --- | --- |
| No. of patients | 223 | 179 | 147 |
| Age, mean (SD), years | 53.8 (17.0) | 43.1 (15.2) | 56.9 (16.4) |
| Gender - Female, No. (%) | 101 (45.3%) | 80 (44.7%) | 66 (44.9%) |
| Berlin classification |  |  |  |
| Mild (%) | 12 (5.4%) | 4 (2.2%) | 7 (4.8%) |
| Moderate (%) | 93 (41.7%) | 42 (23.7%) | 53 (36.6%) |
| Severe (%) | 118 (52.9%) | 131 (75.1%) | 85 (58.6%) |
| Temperature, mean (SD), °C | 38.2 (0.8) | 39.0 (0.8) | 38.2 (0.9) |
| Heart rate, mean (SD), BPM | 116.1 (19.0) | 141.9 (20.5) | 118.3 (24.5) |
| Respiratory rate, mean (SD), breaths/min | 29.1 (9.0) | 39.1 (10.1) | 30.6 (8.2) |
| Systolic blood pressure, mean (SD), mm Hg | 92.7 (16.3) | 83.6 (13.7) | 87.0 (18.0) |
| Glasgow Coma Scale score, mean (SD) | 10.3 (4.1) | 8.8 (4.4) | 8.6 (4.3) |
| Potassium, mean (SD), mEq/L | 3.8 (0.5) | 3.6 (0.5) | 4.1 (0.7) |
| Sodium, mean (SD), mEq/L | 137.2 (4.7) | 136.6 (4.9) | 136.7 (6.0) |
| Glucose, median (IQR), mg/dL | 126.0 (105.0-157.0) | 108.5 (90.0-137.8) | 124.0 (91.0-158.0) |
| Hematocrit, mean (SD), g/dL | 31.5 (5.5) | 29.7 (5.9) | 27.6 (5.1) |
| Creatinine, median (IQR), mg/dL | 0.9 (0.7-1.1) | 0.9 (0.7-1.4) | 2.5 (1.7-4.4) |
| Blood urea nitrogen, median (IQR), mg/dL | 16.0 (11.0-23.5) | 17.0 (11.0-25.8) | 49.0 (35.0-71.0) |
| Bicarbonate, mean (SD), mmol/L | 25.4 (3.8) | 20.3 (5.1) | 17.9 (4.9) |
| Platelets, median (IQR), ×10^9^ /L | 198.5 (124.8-302.2) | 128.0 (72.0-205.0) | 120.0 (61.0-203.0) |
| White blood cell count, median (IQR), ×10^9^ /L | 13.1 (9.2-17.5) | 12.3 (7.2-17.3) | 14.0 (9.2-20.4) |
| Albumin, mean (SD), g/dL | 2.2 (0.6) | 1.9 (0.6) | 2.1 (0.6) |
| Total Bilirubin, median (IQR), mg/dL | 0.7 (0.4-1.2) | 0.8 (0.5-1.8) | 1.0 (0.6-1.8) |
| PaO2, mean (SD), mm Hg | 63.0 (54.0-73.0) | 59.0 (51.0-68.0) | 64.0 (57.0-74.0) |
| PaCO2, mean (SD), mm Hg | 38.0 (34.0-41.0) | 31.0 (28.0-35.0) | 31.0 (26.0-36.8) |
| pH, mean (SD), unit | 7.3 (0.1) | 7.3 (0.1) | 7.3 (0.1) |
| 60 day mortality, No. (%) | 42 (18.8%) | 51 (28.5%) | 51 (34.7%) |
| ICU free days, median (IQR), d | 18.0 (9.0-22.0) | 10.0 (0.0-19.5) | 8.0 (0.0-20.0) |
| Ventilator free days, median (IQR), d | 21.0 (11.5-24.0) | 14.0 (0.0-22.0) | 11.0 (0.0-22.5) |
| IL-6, median (IQR), (pg/ml) | 164.0 (67.0-398.5) | 421.5 (149.5-1447.2) | 246.0 (95.5-939.0) |

Abbreviations: APACHE - Acute Physiology and Chronic Health Evaluation; CABG - Coronary artery bypass graft; COPD - Chronic obstructive pulmonary disease; BPM - beats per minute; IQR - interquartile range; PaO2 - Partial pressure of oxygen; PaCO2 - Partial pressure of carbon dioxide;

Categorical variables are measured by occurrence and percentage. Normally distributed numerical variables are measured by mean and standard deviation, Non-normally distributed numerical variables are measured by median and interquartile range.

Table S9. Clinical characteristics by phenotype in FACTT

| **Characteristic** | **phenotype-I** | **phenotype-II** | **phenotype-III** |
| --- | --- | --- | --- |
| No. of patients | 388 | 313 | 294 |
| Age, mean (SD), years | 50.4 (15.5) | 44.0 (14.5) | 55.0 (16.2) |
| Gender - Female, No. (%) | 184 (47.4%) | 162 (51.8%) | 116 (39.5%) |
| APACHE III Score, mean (SD) | 75.6 (22.8) | 98.9 (27.9) | 113.9 (29.2) |
| Berlin classification |  |  |  |
| Mild (%) | 25 (6.5%) | 16 (5.1%) | 23 (8%) |
| Moderate (%) | 181 (46.9%) | 103 (33%) | 102 (35.3%) |
| Severe (%) | 176 (45.6%) | 193 (61.9%) | 164 (56.7%) |
| Temperature, mean (SD), °C | 38.1 (0.9) | 38.9 (0.9) | 38.1 (1.0) |
| Heart rate, mean (SD), BPM | 114.0 (18.8) | 137.4 (18.9) | 124.5 (20.9) |
| Respiratory rate, mean (SD), breaths/min | 31.1 (9.0) | 39.9 (10.4) | 34.2 (8.8) |
| Systolic blood pressure, mean (SD), mm Hg | 94.6 (17.0) | 84.3 (14.6) | 83.9 (14.8) |
| Glasgow Coma Scale score, mean (SD) | 9.0 (3.9) | 7.3 (3.8) | 7.4 (4.1) |
| Potassium, mean (SD), mEq/L | 3.8 (0.6) | 3.5 (0.5) | 4.1 (0.7) |
| Sodium, mean (SD), mEq/L | 138.0 (5.1) | 137.5 (5.7) | 136.6 (6.6) |
| Glucose, median (IQR), mg/dL | 119.0 (99.0-148.0) | 106.0 (90.0-127.0) | 110.0 (87.0-145.2) |
| Hematocrit, mean (SD), g/dL | 31.6 (6.0) | 28.4 (6.5) | 28.7 (6.5) |
| Creatinine, median (IQR), mg/dL | 0.8 (0.7-1.1) | 0.9 (0.7-1.2) | 2.0 (1.5-2.7) |
| Blood urea nitrogen, median (IQR), mg/dL | 16.0 (11.0-22.0) | 14.0 (10.0-21.0) | 41.0 (26.0-56.0) |
| Bicarbonate, mean (SD), mmol/L | 24.9 (4.5) | 20.0 (5.0) | 17.8 (4.7) |
| Platelets, median (IQR), ×10^9^ /L | 213.0 (145.0-301.0) | 163.0 (93.0-235.0) | 135.5 (71.0-208.5) |
| White blood cell count, median (IQR), ×10^9^ /L | 13.3 (9.2-18.0) | 11.7 (6.6-17.2) | 11.8 (6.5-19.6) |
| Albumin, mean (SD), g/dL | 2.5 (0.7) | 2.1 (0.6) | 2.1 (0.6) |
| Total Bilirubin, median (IQR), mg/dL | 0.6 (0.4-1.1) | 0.9 (0.5-1.8) | 1.0 (0.6-2.1) |
| PaO2, mean (SD), mm Hg | 67.0 (58.0-79.0) | 61.0 (52.0-73.0) | 64.0 (54.0-74.8) |
| PaCO2, mean (SD), mm Hg | 40.0 (35.0-44.0) | 32.0 (27.9-36.0) | 33.0 (29.0-38.8) |
| pH, mean (SD), unit | 7.3 (0.1) | 7.3 (0.1) | 7.2 (0.1) |
| 60 day mortality, No. (%) | 65 (16.8%) | 84 (26.8%) | 119 (40.5%) |
| ICU free days, median (IQR), d | 17.0 (5.0-22.0) | 15.0 (0.0-20.0) | 7.0 (0.0-18.0) |
| Ventilator free days, median (IQR), d | 19.0 (7.0-24.0) | 17.0 (0.0-22.0) | 10.0 (0.0-20.0) |

Abbreviations: APACHE - Acute Physiology and Chronic Health Evaluation; CABG - Coronary artery bypass graft; COPD - Chronic obstructive pulmonary disease; BPM - beats per minute; IQR - interquartile range; PaO2 - Partial pressure of oxygen; PaCO2 - Partial pressure of carbon dioxide;

Categorical variables are measured by occurrence and percentage. Normally distributed numerical variables are measured by mean and standard deviation, Non-normally distributed numerical variables are measured by median and interquartile range.

Table S10. Clinical characteristics by phenotype in SAILS

| **Characteristic** | **phenotype-I** | **phenotype-II** | **phenotype-III** |
| --- | --- | --- | --- |
| No. of patients | 286 | 229 | 230 |
| Age, mean (SD), years | 54.9 (15.8) | 48.4 (16.3) | 58.8 (15.4) |
| Gender - Female, No. (%) | 146 (51.0%) | 136 (59.4%) | 98 (42.6%) |
| APACHE III Score, mean (SD) | 77.0 (22.6) | 99.0 (28.0) | 107.5 (24.3) |
| Berlin classification |  |  |  |
| Mild (%) | 31 (11.9%) | 23 (10.5%) | 31 (14%) |
| Moderate (%) | 131 (50%) | 87 (39.7%) | 114 (51.6%) |
| Severe (%) | 100 (38.2%) | 109 (49.8%) | 76 (34.4%) |
| Temperature, mean (SD), °C | 37.9 (0.8) | 38.6 (0.9) | 37.8 (1.0) |
| Heart rate, mean (SD), BPM | 111.2 (19.4) | 131.8 (22.2) | 113.9 (21.4) |
| Respiratory rate, mean (SD), breaths/min | 29.8 (7.2) | 38.0 (8.4) | 31.6 (7.7) |
| Systolic blood pressure, mean (SD), mm Hg | 90.2 (15.9) | 81.1 (14.7) | 83.6 (14.6) |
| Glasgow Coma Scale score, mean (SD) | 8.6 (3.3) | 7.2 (3.5) | 7.7 (3.3) |
| Potassium, mean (SD), mEq/L | 3.8 (0.6) | 3.5 (0.5) | 4.1 (0.6) |
| Sodium, mean (SD), mEq/L | 138.7 (5.0) | 137.5 (5.6) | 137.0 (5.6) |
| Glucose, median (IQR), mg/dL | 119.5 (102.2-146.8) | 106.0 (90.0-133.0) | 119.0 (97.0-145.0) |
| Hematocrit, mean (SD), g/dL | 31.6 (6.0) | 29.2 (5.8) | 28.6 (6.3) |
| Creatinine, median (IQR), mg/dL | 0.8 (0.7-1.2) | 0.9 (0.7-1.3) | 2.2 (1.6-3.2) |
| Blood urea nitrogen, median (IQR), mg/dL | 16.0 (12.0-24.0) | 16.0 (10.0-24.0) | 42.0 (30.0-60.0) |
| Bicarbonate, mean (SD), mmol/L | 25.4 (4.9) | 20.0 (4.5) | 18.9 (4.6) |
| Platelets, median (IQR), ×10^9^ /L | 218.0 (152.2-277.8) | 141.0 (77.0-215.0) | 128.5 (67.2-224.0) |
| White blood cell count, median (IQR), ×10^9^ /L | 13.0 (8.9-17.1) | 12.9 (7.1-19.5) | 16.2 (9.9-25.0) |
| Albumin, mean (SD), g/dL | 2.4 (0.6) | 2.0 (0.6) | 2.1 (0.6) |
| Total Bilirubin, median (IQR), mg/dL | 0.7 (0.4-1.0) | 0.8 (0.6-1.5) | 1.0 (0.6-1.8) |
| PaO2, mean (SD), mm Hg | 72.0 (61.0-84.0) | 68.0 (57.5-82.0) | 70.0 (62.0-84.8) |
| PaCO2, mean (SD), mm Hg | 42.0 (36.0-48.0) | 32.0 (28.0-37.0) | 34.5 (29.2-39.0) |
| pH, mean (SD), unit | 7.3 (0.1) | 7.3 (0.1) | 7.3 (0.1) |
| 60 day mortality, No. (%) | 65 (22.7%) | 57 (24.9%) | 77 (33.5%) |
| ICU free days, median (IQR), d | 19.0 (6.2-24.0) | 17.0 (0.0-22.0) | 14.0 (0.0-21.8) |
| Ventilator free days, median (IQR), d | 22.0 (11.2-24.0) | 19.0 (1.0-24.0) | 17.0 (0.0-23.0) |

Abbreviations: APACHE - Acute Physiology and Chronic Health Evaluation; CABG - Coronary artery bypass graft; COPD - Chronic obstructive pulmonary disease; BPM - beats per minute; IQR - interquartile range; PaO2 - Partial pressure of oxygen; PaCO2 - Partial pressure of carbon dioxide;

Categorical variables are measured by occurrence and percentage. Normally distributed numerical variables are measured by mean and standard deviation, Non-normally distributed numerical variables are measured by median and interquartile range.

Table S11. Difference in clinical outcomes between phenotypes in three RCTs

(A) Difference in clinical outcomes between phenotypes in ALVEOLI Trail

|  | **phenotype-I** | **phenotype-II** | **phenotype-III** | **p-value*** |
| --- | --- | --- | --- | --- |
| Mortality | 42 (18.8%) | 51 (28.5%) | 51 (34.7%) | 0.004 |
| Ventilator-free days | 21.0 (11.5-24.0) | 14.0 (0.0-22.0) | 11.0 (0.0-22.5) | <0.001 |
| ICU-free days | 18.0 (9.0-22.0) | 10.0 (0.0-19.5) | 8.0 (0.0-20.0) | <0.001 |

* p-values for 3 sample test of independence by Chi-squared test and Kruskal–Wallis test

(B) Difference in clinical outcomes between phenotypes in FACTT Trail

|  | **phenotype-I** | **phenotype-II** | **phenotype-III** | **p-value** |
| --- | --- | --- | --- | --- |
| Mortality | 65 (16.8%) | 84 (26.8%) | 119 (40.5%) | <0.001 |
| Ventilator-free days | 19.0 (7.0-24.0) | 17.0 (0.0-22.0) | 10.0 (0.0-20.0) | <0.001 |
| ICU-free days | 17.0 (5.0-22.0) | 15.0 (0.0-20.0) | 7.0 (0.0-18.0) | <0.001 |

* p-values for 3 sample test of independence by Chi-squared test and Kruskal–Wallis test

(C) Difference in clinical outcomes between phenotypes in SAILS Trail

|  | **phenotype-I** | **phenotype-II** | **phenotype-III** | **p-value** |
| --- | --- | --- | --- | --- |
| Mortality | 65 (22.7%) | 57 (24.9%) | 77 (33.5%) | 0.02 |
| Ventilator-free days | 22.0 (11.2-24.0) | 19.0 (1.0-24.0) | 17.0 (0.0-23.0) | <0.001 |
| ICU-free days | 19.0 (6.2-24.0) | 17.0 (0.0-22.0) | 14.0 (0.0-21.8) | <0.001 |

* p-values for 3 sample test of independence by Chi-squared test and Kruskal–Wallis test

References

1. Pollard TJ, Johnson AE, Raffa JD, Celi LA, Mark RG, Badawi O. The eICU Collaborative Research Database, a freely available multi-center database for critical care research. Scientific data. 2018 Sep 11;5:180178.
2. Azur MJ, Stuart EA, Frangakis C, Leaf PJ. Multiple imputation by chained equations: what is it and how does it work?. International journal of methods in psychiatric research. 2011 Mar;20(1):40-9.
3. White IR, Royston P, Wood AM. Multiple imputation using chained equations: issues and guidance for practice. Statistics in medicine. 2011 Feb 20;30(4):377-99.
4. Ankerst M, Breunig MM, Kriegel HP, Sander J. OPTICS: ordering points to identify the clustering structure. ACM Sigmod record. 1999 Jun 1;28(2):49-60.
5. Maaten LV, Hinton G. Visualizing data using t-SNE. Journal of machine learning research. 2008;9(Nov):2579-605.
6. Tibshirani R, Walther G, Hastie T. Estimating the number of clusters in a data set via the gap statistic. Journal of the Royal Statistical Society: Series B (Statistical Methodology). 2001;63(2):411-23.
7. Mohajer M, Englmeier KH, Schmid VJ. A comparison of Gap statistic definitions with and without logarithm function. arXiv preprint arXiv:1103.4767. 2011 Mar 24.
8. Monti S, Tamayo P, Mesirov J, Golub T. Consensus clustering: a resampling-based method for class discovery and visualization of gene expression microarray data. Machine learning. 2003 Jul 1;52(1-2):91-118.
9. Wilkerson MD, Hayes DN. ConsensusClusterPlus: a class discovery tool with confidence assessments and item tracking. Bioinformatics. 2010 Jun 15;26(12):1572-3.
10. Wang R, Lagakos SW, Ware JH, Hunter DJ, Drazen JM. Statistics in medicine—reporting of subgroup analyses in clinical trials. New England Journal of Medicine. 2007 Nov 22;357(21):2189-94.
